# Incidence of microvascular dysfunction is increased in hyperlipidemic mice, reducing cerebral blood flow and impairing remote memory

**DOI:** 10.3389/fendo.2024.1338458

**Published:** 2024-02-26

**Authors:** Luis Daniel Hernandez Torres, Flavia Rezende, Eva Peschke, Olga Will, Jan-Bernd Hövener, Frauke Spiecker, Ümit Özorhan, Josephine Lampe, Ines Stölting, Zouhair Aherrahrou, Carsten Künne, Kristina Kusche-Vihrog, Urte Matschl, Susanne Hille, Ralf P. Brandes, Markus Schwaninger, Oliver J. Müller, Walter Raasch

**Affiliations:** ^1^Institute for Experimental and Clinical Pharmacology and Toxicology, University of Lübeck, Lübeck, Germany; ^2^Institute for Cardiovascular Physiology, Faculty of Medicine, Goethe-University Frankfurt, Frankfurt, Germany; ^3^DZHK (German Center for Cardiovascular Research) Partner Site Rhine-Main, Germany; ^4^Section Biomedical Imaging, Molecular Imaging North Competence Center (MOIN CC), Department of Radiology and Neuroradiology, Universitätsklinikum Schleswig-Holstein (UKSH), Kiel University, Kiel, Germany; ^5^Institute for Cardiogenetics, University Lübeck; University of Lübeck, Lübeck, Germany; ^6^DZHK (German Centre for Cardiovascular Research), Partner Site Hamburg/Kiel/Lübeck, Germany; ^7^Department of Cardiac Development and Remodeling, Max Planck Institute for Heart and Lung Research, Bad Nauheim, Germany; ^8^Institute for Physiology, University Lübeck, Lübeck, Germany; ^9^Department Virus Immunology, Heinrich Pette Institute, Leibniz Institute for Experimental Virology, Hamburg, Germany; ^10^Department of Internal Medicine III, University Hospital Schleswig-Holstein, Kiel, Germany; ^11^CBBM (Centre for Brain, Behavior and Metabolism), University of Lübeck, Lübeck, Germany

**Keywords:** atherosclerosis AAV-PCSK9^DY^ mouse model, cognitive dysfunction, cerebral blood flow, hyperlipidemia, pericytes, patchy blood-brain barrier leaks

## Abstract

**Introduction:**

The development of cognitive dysfunction is not necessarily associated with diet-induced obesity. We hypothesized that cognitive dysfunction might require additional vascular damage, for example, in atherosclerotic mice.

**Methods:**

We induced atherosclerosis in male C57BL/6N mice by injecting AAV-PCSK9^DY^ (2x10^11^ VG) and feeding them a cholesterol-rich Western diet. After 3 months, mice were examined for cognition using Barnes maze procedure and for cerebral blood flow. Cerebral vascular morphology was examined by immunehistology.

**Results:**

In AAV-PCSK9^DY^-treated mice, plaque burden, plasma cholesterol, and triglycerides are elevated. RNAseq analyses followed by KEGG annotation show increased expression of genes linked to inflammatory processes in the aortas of these mice. In AAV-PCSK9^DY^-treated mice learning was delayed and long-term memory impaired. Blood flow was reduced in the cingulate cortex (-17%), caudate putamen (-15%), and hippocampus (-10%). Immunohistological studies also show an increased incidence of string vessels and pericytes (CD31/Col IV staining) in the hippocampus accompanied by patchy blood-brain barrier leaks (IgG staining) and increased macrophage infiltrations (CD68 staining).

**Discussion:**

We conclude that the hyperlipidemic PCSK9^DY^ mouse model can serve as an appropriate approach to induce microvascular dysfunction that leads to reduced blood flow in the hippocampus, which could explain the cognitive dysfunction in these mice.

## Introduction

Alzheimer disease (AD) and vascular dementia represent age-related cognitive disorders of major impact in public health. Both have a multifactorial origin, with cardiovascular risk factors playing a large role (1). Vascular dementia includes cerebral small-vessel diseases (CSVD), a cluster of vessel abnormalities in the brain that can produce small subcortical infarcts, prominent perivascular spaces, microbleeds, and atrophy (2). Vascular risk factors, that may promote development of metabolic syndrome (MetS), contribute to the changes in small vessels, such as inflammatory responses, hypoperfusion, oxidative stress, disrupted blood-brain barrier, and, ultimately, cognitive dysfunction and vascular dementia (3, 4). MetS is defined as a cluster of the four cardinal symptoms hyperlipidemia, insulin resistance, obesity and hypertension, thus leading to heart disease, diabetes, stroke and other health problems. MetS is often associated with atherosclerosis, a condition that affects the integrity of the large blood vessels and compromises the blood supply to most organs, including the brain. The effects of dyslipidemia associated with atherosclerosis on the large vessels is well established, but its impact on the small vessels of the brain and the occurrence of vascular dementia is poorly understood (5).

For this reason, interest in the pathophysiology of CSVD in the context of vascular dementia and AD is increasing. In AD patients the density of string vessels in the brain is higher. These vessels consist of empty basement membrane tubes that are connected to blood vessels but lack a lumen or endothelial cells (6). String vessels are not the only vascular abnormality found in dementias. In addition to reduced density of small cortical vessels, there are more tortuous vessels, perivascular spaces are enlarged, and microaneurysms develop in brains of patients with vascular dementia (7).

In this study we investigated the burden of cognitive dysfunction and small cerebral vessel changes in C57Bl/6N mice which received a single injection of the AAV-PCSK9^DY^ vector (adeno-associated virus-8-mediated overexpression of pro-protein convertase subtilisin/kexintype 9^DY^) and which were fed with a high-cholesterol Western diet (WD). PCSK9 decreases hepatic uptake of LDL by increasing endosomal and lysosomal degradation of LDL receptors. Thus, mice lacking PCSK9 protein have low plasma LDL cholesterol levels and are protected from developing atherosclerosis (8). PCSK9 inhibition is, therefore, a rational therapeutic target in treating patients suffering from atherosclerosis (9). In contrast, the single injection of the PCSK9^DY^ mutant results in hypercholesterolemia and accelerated atherosclerosis formation (10, 11) accompanied by the development of endothelial dysfunction of the aorta (12), while it is not yet known whether the integrity and functionality of the cerebral vessels in particular is also deteriorated. We deliberately used the PCSK9^DY^/WD based atherosclerosis model in contrast to the ApoE or LepR ko-models, as this model does not require any breeding effort and low (cost) effort in terms of the 3R rule and we intend to continue this approach in follow-up (therapy) studies by also using transgenic mouse lines.

We here found an increased incidence of string vessels and pericytes in the hippocampus that was accompanied by patchy blood-brain barrier leaks and increased macrophage infiltrations. These changes were associated with impaired memory consolidation and reduced blood flow in the hippocampus, suggesting that hippocampus-specific microvascular dysfunction is associated with reduced blood flow and, moreover, with cognitive dysfunction in AAV-PCSK9^DY^+WD mice.

## Methods

### AAV vector production and purification

AAV serotype 8 vectors for expression of the murine D377Y-PCSK9 cDNA (AAV-PCSK^9^) were produced as previously described (13) by using the two-plasmid method and cotransfecting AAV/D377Y-mPCSK9 (gift from Jacob Bentzon; Addgene plasmid # 58376) (10) together with the helper plasmid pDP8 (14) in HEK293T cells using polyethylenimine (Sigma Aldrich). AAV vectors were purified using iodixanol step gradients and titrated as previously described (15). Here, this vector is denoted as AAV-PCSK9^DY^.

### Animals

All animal care and experimental procedures were conducted in accordance with the NIH guidelines for care and use of laboratory animals and were approved by the local animal ethics committee (Ministerium für Landwirtschaft, ländliche Räume, Europa und Verbraucherschutz des Landes Schleswig-Holstein, Germany) under the application number 44-6/21. The results of all studies involving animals are reported in accordance with the ARRIVE guidelines (16). The group sizes of n=10 per condition were assessed by a power analysis (corrected α = 0.01, power 80%) by considering cerebral blood flow (CBF) (17). In total, 20 10- to 11-week-old male C57BL/6N mice (Janvier Labs, Germany) were used for this study. All mice were kept in groups of 2-3 individuals randomized by weight. Mice had ad libitum access to standard chow diet for a 2-week habituation period before starting the study and receiving the AAV-PCSK9^DY^ injections.

### Protocol

Mice received AAV-PCSK9^DY^ injections (2×10^11^ VG in 100 µl) or the same volume of saline via the tail vein at an age of 10 to 11 weeks. Afterwards, AAV-PCSK9^DY^-injected mice were fed a WD (WD, EF TD88137 mod. +1.25% cholesterol, Sniff Spezialdiäten GmbH, Metabolizable Energy 19.1 MJ/kg, 42kJ% fat, 15kJ% protein, 43kJ% carbohydrates; Crude Nutrients [%], crude protein (N x 6.25) 17.3, crude fat 21.1, crude fibre 5.0, crude ash 4.2, starch 13.4, sugar 34.1, N free extracts 48.7, cholesterol 12,950 mg/kg) for 3 months, while the saline-treated controls received standard maintenance chow (12). Body weight was monitored and body composition was determined at week 15 using the nuclear magnetic resonance (NMR) method (Minispec BCA analyzer, LF-110, Bruker) 2 h after the mice were transferred to the NMR room to acclimatize to the environment. For the measurements, mice were put into a restrainer, which was then placed in the analyzer (12, 17). Cognition of mice was determined from week 11 to week 14 by open-field (OF), object place recognition (OPR), elevated plus maze (EPM), as well as Barnes maze (BM) tests as previously described (12, 17). At the end of the study, CBF was determined by ASL-MRI at week 16 (17). Immediately after ASL-MRI measurements, blood samples (non-fasted) were collected by cardiac puncture. Mice were sacrificed and transcardially perfused with Ringer-heparin solution. Aortic arch, descending aorta, and brain were snap frozen in liquid nitrogen and stored at -80°C for further analyses. The protocol is summarized in **Supplementary Figure S1**.

### Behavior tests

The OF test was performed at week 11, the OF apparatus being 370 mm x 370 mm and light set to 19 lux. Mice were placed in the center of apparatus and allowed to freely explore for 10 min; the test was repeated for 3 days. Time spent in the center and immobile time were measured here.

EPM was performed at week 12 with a plus sign-shaped apparatus. The maze consisted of a plastic cross, placed 40 cm above the floor. The two opposing, closed arms of the EPM apparatus (30 cm x 5 cm x 16 cm) were dimly illuminated by a light bulb at 50 lux. The two opposing open arms of the EPM (30 cm x 5 cm x 0.5 cm) were brightly illuminated by a light bulb at 250 lux. The four arms were connected by a central platform (5 cm x 5 cm, illuminated at 100 lux). At the beginning of the test, the mouse was placed on the central platform, facing one of the closed arms and allowed to explore for 5 min. Immobile time, exploration events in open arm, and time spent in open or closed arm were measured.

OPR test was performed at week 11 by using an open box (370 mm x 370 mm, light set to 19 lux). Mice were habituated for 10 min on 3 consecutive days and, on the testing day, also placed in the center and allowed to explore two identical objects placed in two of the corners for 10 min. After 1 h, mice were allowed to explore the apparatus, in which one of the objects was relocated into another corner, for 5 min. The interaction with the displaced object was evaluated as preference index.

For BM testing at week 13-14, the mice were placed in the center to explore the brightly lit (300 lux) platform (90-cm diameter with 20 holes) for a maximum of 180 s to find the dark escape hole. Mice were gently led to the escape hole if they could not find it. During the training phases (for 5 consecutive days), the test was repeated 4 times for each mouse each day with an inter-trial interval of 15 min. approximately. Long-term and remote memory tests were performed at day 6 and 17, respectively; on these days the escape hole was closed and the maze orientation was turned 180°, then mice were allowed to explore for 90s. During all trials, the video analysis was made by semi-automatic measurements of primary latency (time until the escape hole was reached the first time), time mobile (the time the mice were mobile in the given 90sec during the experiment) and manual measurements of primary errors (number of head dips into wrong holes until first contact with the escape hole). Search strategy were categorized as defined by Harrison et al., 2006 as direct, serial or random and we determined the search strategy preference to find the escape hole (18). Example of search strategy category can be found in **Supplementary Figure S2**. In addition, the following secondary parameters were measured: mean speed distance travelled (distance that the mice has moved until the end of the test), secondary latency (during the learning phase, this is the time the mice required to solve the test, from second 0 until they enter into the escape hole), exit interaction^time^ (this is the total time the mice had been exploring the escape hole, regardless of whether the mice enters or not), and exit interaction^events^ (this is the total number of interactions with the escape hole, regardless of whether the mice enters or not). All procedures were recorded and analyzed with ANY-maze Video Tracking System ver. 4.114 (©1999-2013 Stoelting Co).

### Arterial spin labeling MRI

According to previous reports (17), the mice were scanned at a 7-Tesla small-animal MRI scanner (BioSpec, Bruker, Germany) using an 86-mm quadrature volume transmit-receive coil and 2x2 channel receive-only surface coil. Prior to the ASL sequence, a T2-weighted spin-echo sequence (TR=2500ms, TE=33ms, FOV=20x20mm², matrix=256x256, slice thickness=0.7mm) and 3D time-of-flight (TOF) (TR=12ms, TE=3ms, FOV=20x20mm², matrix=256x256, slab thickness=15mm) were performed. Perfusion was measured by continuous arterial spin labeling (CASL) with echo-planar imaging (EPI) readout (TR=4000 ms, TE=16 ms, FOV=25x25 mm², matrix=96x96, slice thickness=1mm). The mean inversion efficiency value was calculated in the carotids; thus, the result of the CASL-EPI is an absolute CBF map. T2-weighted image analysis for tissue perfusion was performed with ImageJ (U. S. National Institutes of Health, Bethesda, Maryland, USA). TOF angiography was performed in the brain with slices in transverse orientation and images were acquired in cross sections of the internal carotid artery (ICA). All images were analyzed with Fiji ImageJ.

### Histological analysis

For all atherosclerotic plaque analyses, investigators were blinded to the viral load. As described previously (12, 19, 20), serial cross sections (8- to 10-μm thick) were obtained, starting below the aortic root to the proximal aorta, below the aortic arch. The sections were stained using Oil Red-O (ORO) and the mean atherosclerotic lesion area was calculated from 8-10 sections at 40-μm intervals, starting at the appearance of at least two aortic valves until the aortic valves disappeared. Images were acquired using a Keyence microscope (BZ-X800). Sections were manually cropped using GIMP software, version 2.6 (The GIMP Development Team) to yield aortic root areas (12). Areas of lesions and ORO-positive regions were determined using an in-house Python script (available on request). In brief, the Python package OpenCV (https://pypi.org/project/opencv-python/) was utilized to process images and to determine lesions based on color thresholds (e.g., reddish pixel for ORO). The ratio of ORO-positive lesions in each animal was determined as the percentage lesion area, normalized to the total area of the aorta.

### RNA sequencing

RNA sequencing (RNAseq) analysis was performed in the descending aorta. Aortic tissue was isolated, trimmed from fat, and snap frozen in liquid nitrogen. For the RNAseq analyses, the aortic segments of 2 mice were pooled and a total of 4 samples were analyzed. This means that the results represent 8 of the 10 mice used each group. Total RNA was isolated with the RNA Mini Kit from Bio& SELL (Nuremberg, Germany) combined with on-column DNase digestion (DNase-Free DNase Set, Qiagen) to avoid genomic DNA contamination. RNA and library preparation integrity were verified with LabChip Gx Touch 24 (Perkin Elmer). As input for VAHTS Stranded mRNA-seq Library preparation, 500 µg of total RNA was used following the manufacturer’s protocol (Vazyme). Sequencing was performed on a NextSeq2000 instrument (Illumina) with 1x72-bp single-end setup. The resulting raw reads were assessed for quality, adapter content, and duplication rates with FastQC (RRID : SCR_014583) (Andrews Simon: Andrews Simon: FastQC: a quality control tool for high-throughput sequence data. Available online at http://www.bioinformatics.babraham.ac.uk/projects/fastqc).

### RNA sequencing analysis

Trimmomatic version 0.39 was employed to trim reads after a quality drop below a mean of Q15 in a window of 5 nucleotides and keeping only filtered reads longer than 15 nucleotides (21). Reads were aligned versus Ensembl mouse genome version mm10 (Ensembl release 101) with STAR 2.7.10a (22). Aligned reads were filtered to remove duplicates with Picard 2.27.4 (Picard: A set of tools in Java for working with next-generation sequencing data in the BAM format), multi-mapping, ribosomal, or mitochondrial reads. Gene counts were established with featureCounts 2.0.4 by aggregating reads overlapping exons on the correct strand, excluding those overlapping multiple genes (23). The raw count matrix was normalized with DESeq2 version 1.36.0 (24). Contrasts were created with DESeq2 based on the raw count matrix. Genes were classified as significantly differentially expressed at average count > 5, multiple testing adjusted p-value < 0.05, and -0.585 < log2FC > 0.585. The Ensemble annotation was enriched with UniProt data (Activities at the Universal Protein Resource (UniProt)).

### Immunofluorescent staining

Brain immunofluorescence was performed on 20-μm-thick frozen sections. Brain sections were fixed with methanol and blocked in 1% bovine serum albumin (BSA) in phosphate-buffered saline (PBS) for 1 h. For CD31-, Col IV-, CD68- and PDFGrß-staining, sections were incubated in 1% BSA primary antibodies overnight (**Supplementary Table S1**). Then, sections were washed in PBS and incubated in DAPI (1ug/ml), and secondary antibodies (**Supplementary Table S2**) for 1 h. Immunofluorescent images were analyzed by considering 10 randomly selected different regions of interest in a blinded manner in hippocampus and amygdala. String vessels and pericytes were counted manually; the string vessels were considered as thin Col IV-positive and CD31-negative vessels, while pericytes include stuby Col IV-positive and CD31-negative vessels. In this analysis, stuby structures around DAPI-positive nuclei were not counted as pericytes. Pericyte analysis was confirmed by PDGFrβ/Col IV immunostaining, the number of pericytes was measured manually. The CD31/Col IV ratio and IgG/Col IV length ratio, was calculated with ImageJ as the area ratio of CD31-positive area over Col IV-positive area. Patchy blood-brain barrier leaks were detected with the IgG-positive areas outside of Col IV-positive vessels. These patchy blood-brain barrier leaks were counted manually and the area of lesion was measured with ImageJ. IgG deposition in the posterior cerebral artery was calculated as ratio of the length of IgG-positive length over the Col IV-positive length. CD68-positive perivascular macrophages were counted manually in the posterior cerebral artery defined by DAPI staining.

### Cholesterol and triglyceride analyses

Plasma was prepared by centrifugation. Plasma concentrations of total cholesterol (TC) and triglycerides (TG) were analyzed in the mice as described previously (19) to determine whether an increasing viral load plus WD affected lipid profiles.

### Blood cytokine analysis

Plasma concentrations of diverse adipocytokines were measured in 10 µL of sample by immunosorbent assays according to the manufacturer’s instructions using the Bio-Plex 200 platform and the Milliplex mouse metabolic magnetic bead panel kit MMHMAG-44K.mouse (amylin active, C-peptide 2, ghrelin, GIP, GLP-1 active or GLP-1 total, glucagon, IL-6, insulin, leptin, MCP-1, PP, PYY, resistin, secretin, and TNF), and MHSTCMAG-70K (GM-CSF, IFNγ, IL-1α, IL-1β, IL-2, IL-4, IL-5, IL-6, IL-7, IL-10, IL-12 (p70), IL-13, IL-17A, KC/CXCL1, LIX, MCP-1, MIP-2, TNF).

### Statistical analysis

GraphPad Prism 8.0 (La Jolla, USA) was used for statistical analysis. All data were checked for outliers with Rout (Q=1%) test and tested for Gaussian distribution and variance homogeneity with D’Agostino & Pearson test. A two-tailed Student’s t-test was used for comparing different groups, assuming a Gaussian distribution and variance homogeneity. In case of no variance homogeneity, a Welch correction was used. Alternatively, if Gaussian distribution was not given, we used the Mann-Whitney test. A p-value <0.05 was considered statistically significant. Strategy use per day was expressed as percentage of the three strategies (direct, serial, mixed) used within the 5 daily trials. Comparisons between conditions were conducted using Fisher’s exact chi quadrat test (SPSS Statistics 27, IBM, USA). To analyze the change in strategy used across days1-5, Friedman tests were applied (SPSS Statistics 27, IBM, USA). To analyze whether different search strategies differs at d5 between both groups we used Mann-Whitney testing (SPSS Statistics 27, IBM, USA). In box graphs (representing the 25th to 75th percentiles) with whiskers (representing the maximum and minimum values), both the individual data and the medians are presented. In the line graphs, means ± SDs are depicted. Correlation analyses were performed by a one-sided Pearson test.

## Results

### PCSK9^DY^+WD mice developed obesity and atherosclerosis

**Figure 1:**
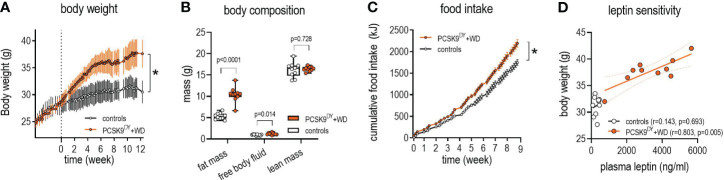
Growth in C57Bl6 mice that received a single AAV-PCSK9^DY^ (2x10^11^ vg) injection plus western diet. Controls only received chow diet. Values are depicted in line graphs as means ± SD. A 2-way ANOVA was calculated considering the factors time, diet, and interaction, showing significances for body weight (**A**: time: F=42.6, P<0.0001, diet F=1843, P<0.0001, interaction F=8.4, P<0.0001), and food intake (**C**: time: F=3125, P<0.0001, diet F=51.5, P<0.0001, interaction F=35.0, P<0.0001). The median is depicted for body composition **(B)** in box blots; the box extends from the 25th to 75th percentiles and the whiskers go down to the smallest value and up to the largest. A t-test was calculated with or without Welsh correction (dependent on variance homogeneity) when values showed Gaussian distribution. n=10 each group. For determination leptin sensitivity **(D)** the correlation between plasma leptin and body weight at final day was calculated by Pearson. *p<0.05.

**Figure 2:**
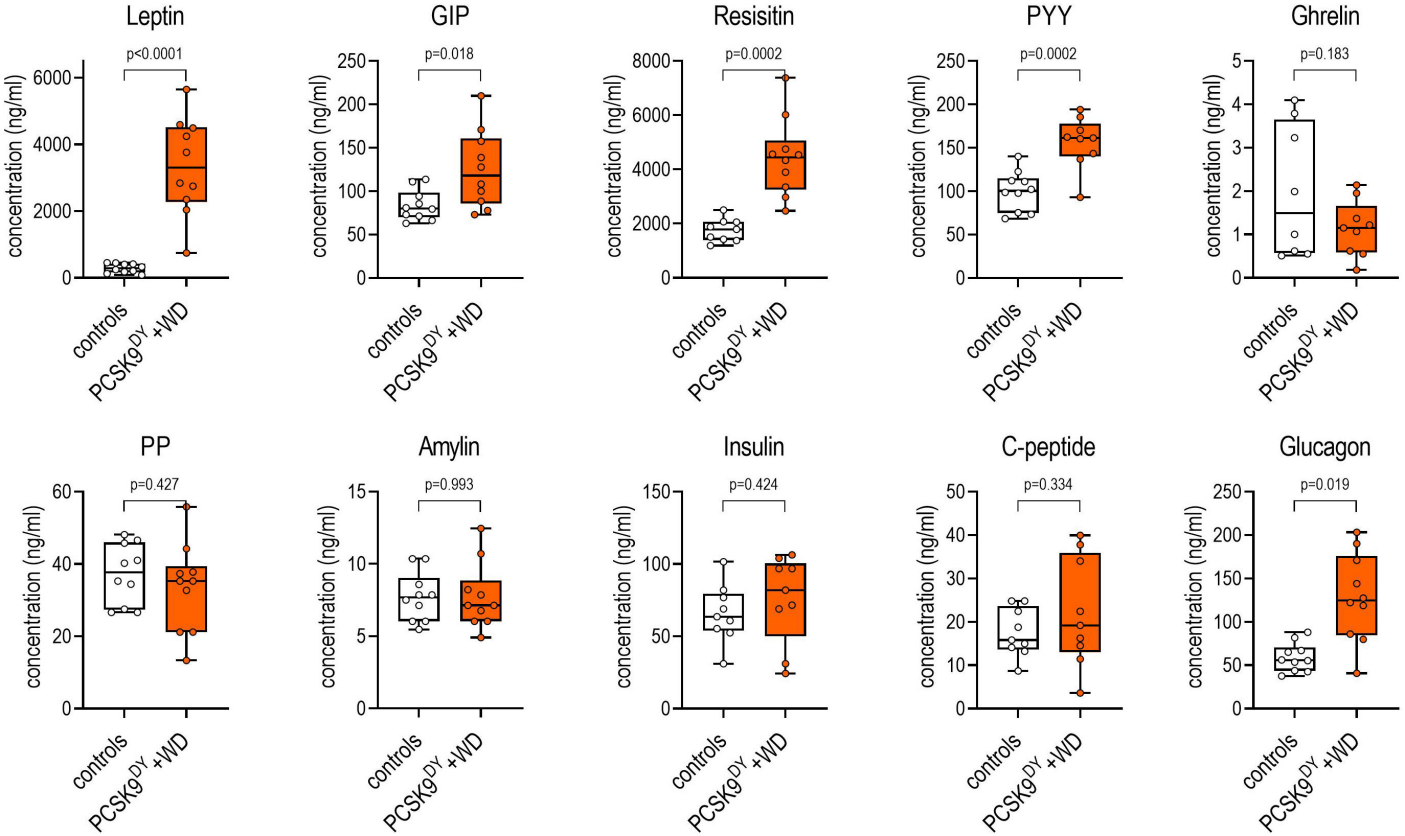
Plasma concentrations of various hormones in C57Bl6 mice that received a single AAV-PCSK9^DY^ (2x10^11^ vg) injection plus western diet. Controls only received chow diet. The median is depicted in box blots; the box extends from the 25th to 75th percentiles and the whiskers go down to the smallest value and up to the largest. A t-test was calculated with or without Welsh correction (dependent on variance homogeneity) when values showed Gaussian distribution. n=10 each group.

We first characterized the development of obesity and atherosclerosis in the PCSK9^DY^+WD mouse model. The final body weight of PCSK9^DY^-treated mice increased by 21% on WD compared to mice fed standard chow (37.7 ± 2.5 vs. 31.2 ± 1.8g, p<0.0001, **Figure 1A**). The NMR analysis indicated that body weight increase was associated with an increase in fat mass (+96%) with no change in lean mass compared to controls. In addition, NMR scans revealed increased free body fluid (+26%, **Figure 1B**). The increased calorie intake (+23%) in the PCSK9^DY^+WD mice caused obesity (**Figure 1C**). PCSK9^DY^+WD mice developed leptin resistance as indicated by the positive correlation between plasma leptin and body weight (**Figure 1D**). The development of obesity in mice fed WD was also reflected by the significantly increased plasma levels of the hormones leptin, peptide YY (PYY), resistin, and glucose-dependent insulinotropic polypeptide (GIP) (**Figure 2**). However, it is unclear as to whether the mice also developed a glucose tolerance disorder as we could not detect any changes in plasma insulin or plasma C-peptide (**Figure 2**).

**Figure 3:**
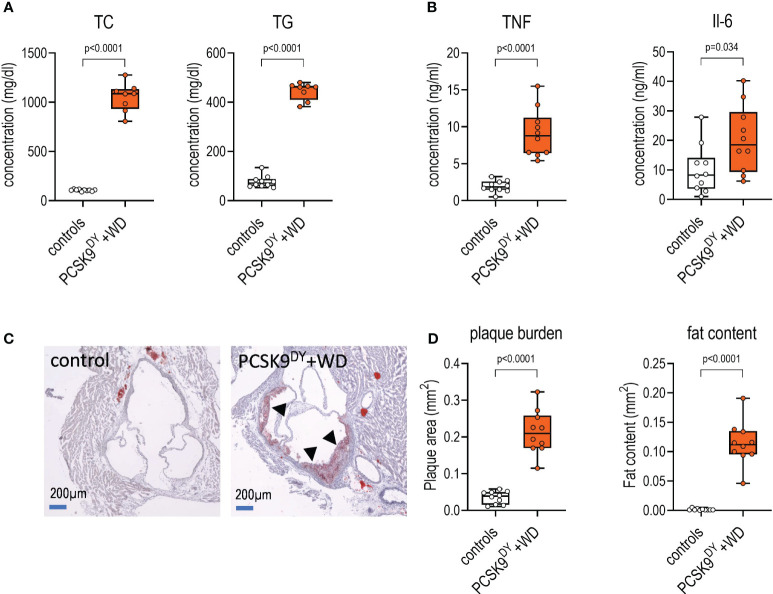
Development of atherosclerotic lesions in mice after PCSK9^DY^ injection plus high-cholesterol Western diet (WD) compared to controls fed chow. **(A)** Plasma concentration of total cholesterol (TC) and triglycerides (TG); **(B)** Plasma concentration of TNF and IL6; **(C)** exemplary aortic segments of controls and PCSK9^DY^+WD-treated mice upon Oil Red O staining (the reddish colorations marked with arrows are plaques); **(D)** quantitative evaluation of plaque and fat content. The median is depicted in box blots; the box extends from the 25th to 75th percentiles and the whiskers go down to the smallest value and up to the largest. A t-test was calculated with or without Welsh correction (dependent on variance homogeneity) when values showed Gaussian distribution. When values did not show Gaussian distribution, Mann-Whitney test was calculated; n=10 each group.

**Figure 4:**
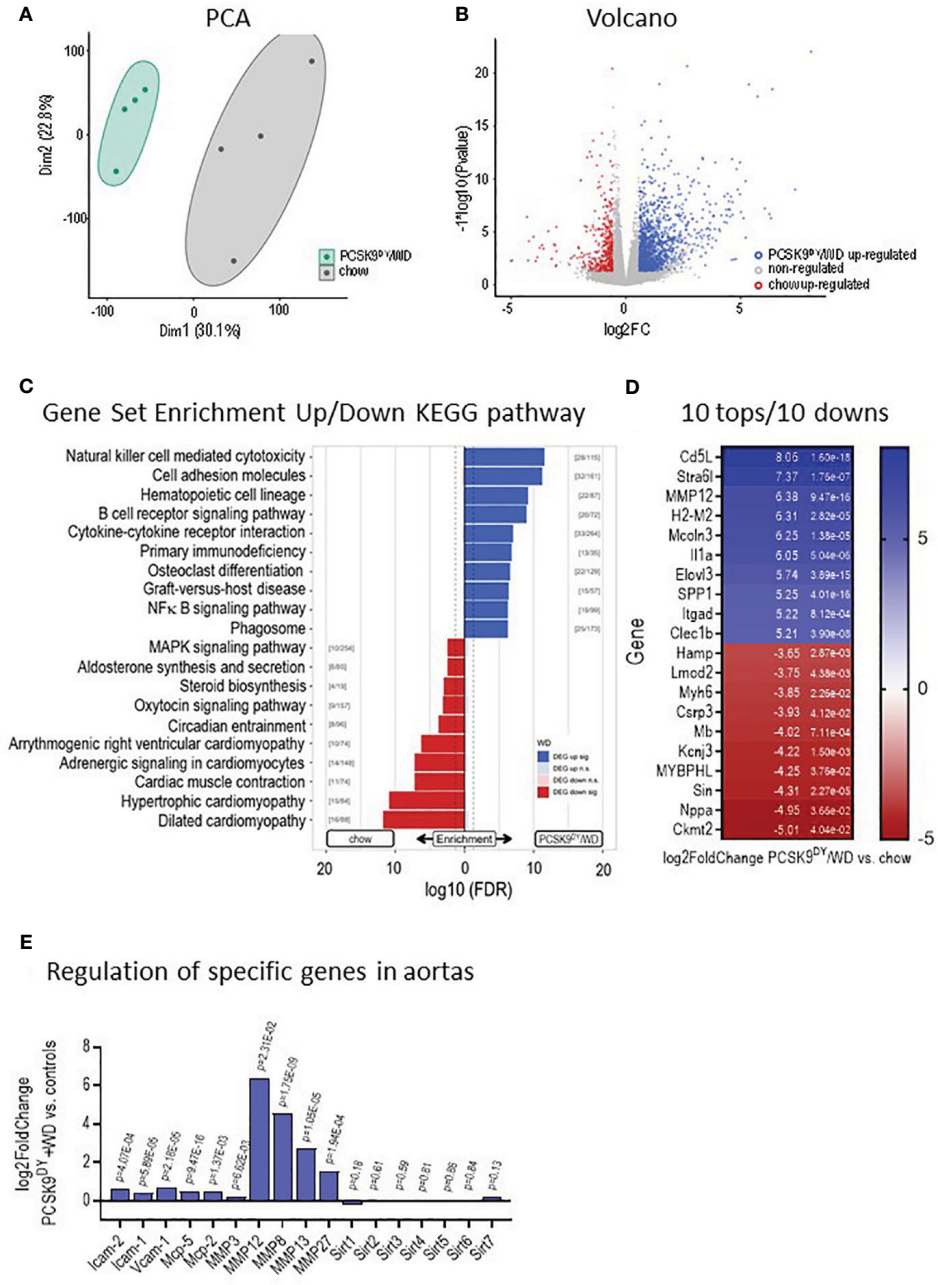
Development of atherosclerotic lesions in mice after PCSK9^DY^ injection plus high-cholesterol Western diet (WD) compared to controls fed chow. **(A)** Principle component analyses (PCA), **(B)** Volcano blots following RNAseq analyses of aortic segments; **(C)** Gene set enrichment up- and downregulation following KEGG analyses indicate upregulation of inflammation pathways in PCSK9^DY^+WD-treated mice; **(D)** 10 tops and 10 downs in regulated genes; **(E)** Regulation of specific genes in aortas of PCSK9^DY^+WD-treated mice compared to controls, n=4.

Plasma concentrations of TC and TG were increased 10- and 6-fold, respectively, in PCSK9^DY^+WD mice as compared to controls (**Figure 3A**). Moreover, plaque burden and lipid content in the plaques were clearly visible in aortas of PCSK9^DY^+WD mice, but not in controls (**Figures 3C, D**). These findings clearly show that atherosclerosis had developed in PCSK9^DY^+WD mice. In addition, elevated plasma levels of TNF (5-fold) and IL-6 (2-fold) revealed systemic inflammation (**Figure 3B**). Other cytokines (IL-10, IL-12, GMCSF, and LIX) were not significantly different between the two treatment groups (**Supplementary Figure S3**). Remarkably, the genes expressed in aortic tissue in the two groups differed significantly (**Supplementary Figure S4**) as shown in the principal component analysis (PCA, **Figure 4A**) and a Volcano blot (**Figure 4B**). Importantly, RNAseq analyses in thoracic aortic segments point to an inflammatory response. KEGG analysis annotate the differentially expressed genes in PCSK9^DY^+WD mice to various, inflammatory pathways (e.g., natural killer cell-mediated cytotoxicity, cell adhesion molecules, hematopoietic cell lineage, B-cell receptor signaling pathway, cytokine-cytokine receptor interaction, and NF-κB signaling pathway) (**Figure 4C**). The most prominent genes upregulated in the PCSK9^DY^+WD mice were Cd5L, Stra6l, MMP12, H2-M2, Mcoln3, Il1a, Elovl3, SPP1, Itgad, and Clec1b (**Figure 4D**), the most significantly down-regulated genes Hamp, Lmod2, Myh6, Csrp3, Mb, Kcnj3, MYBPHL, Sin, Nppa, and Ckmt2 (**Figure 4D**) (25). Moreover, we detected upregulation of genes that are members of the matrix metalloproteinase family (MMP), vascular cell adhesion molecule (V-CAM), intercellular adhesion molecule (I-CAM), and the monocyte chemoattractant protein (MCP) families in aortas of PCSK9^DY^-positive mice, while sirtuins (Sirt1-Sirt7) were rather not regulated (**Figure 4E**).

### Remote memory is impaired in PCSK9^DY^+WD mice

**Figure 5:**
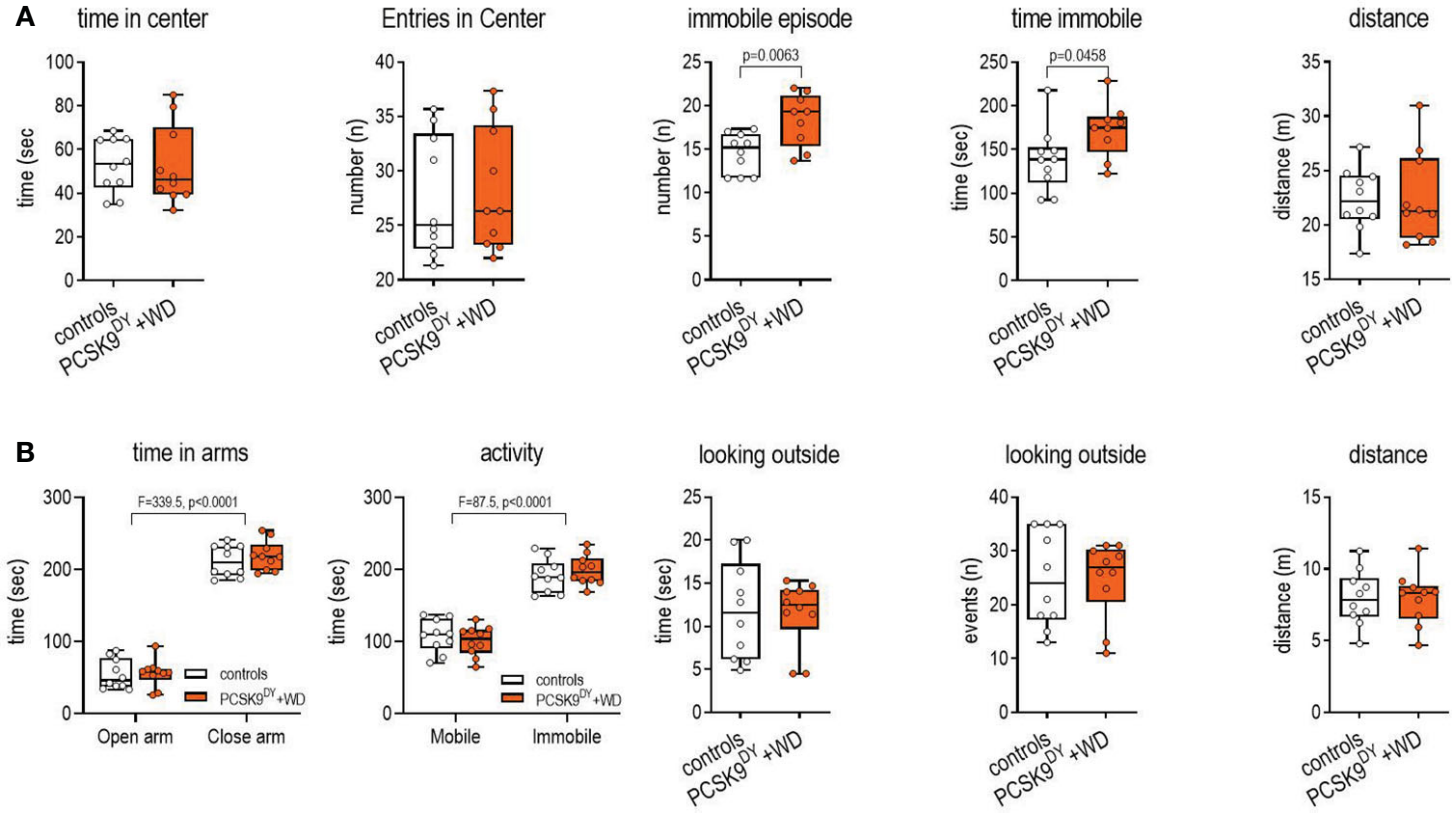
Open-field **(A)** and elevated plus maze **(B)** tests to determine anxiety in C57Bl/6 mice that received a single AAV-PCSK9^DY^ (2x10^11^ vg) injection plus Western diet while controls only received chow diet. The median is depicted in box blots; the box extends from the 25th to 75th percentiles and the whiskers go down to the smallest value and up to the largest. A t-test was calculated with or without Welsh correction (dependent on variance homogeneity) when values showed Gaussian distribution. When values did not show Gaussian distribution Mann-Whitney test was calculated; n=10 each group.

We did not detect anxiety-like behavior in PCSK9^DY^+WD mice with the OF test (**Figure 5A**). The number of entries to the center area or the time spent in the center area or in the distance traveled in the test did not differ between controls and PCSK9^DY^+WD mice. Only the time and number of immobility events was higher in PCSK9^DY^+WD mice, which might be associated with obesity and less with anxious behavior. Furthermore, the EPM test did not show signs of anxiety-like behavior in the mice (**Figure 5B**): entry into the closed and open arms was similar and no differences were found in the time spent in the closed or open arms. When the animals were in the open arms, both groups demonstrated the same exploratory behavior in looking outside of the maze and those events lasted the same time.

**Figure 6:**
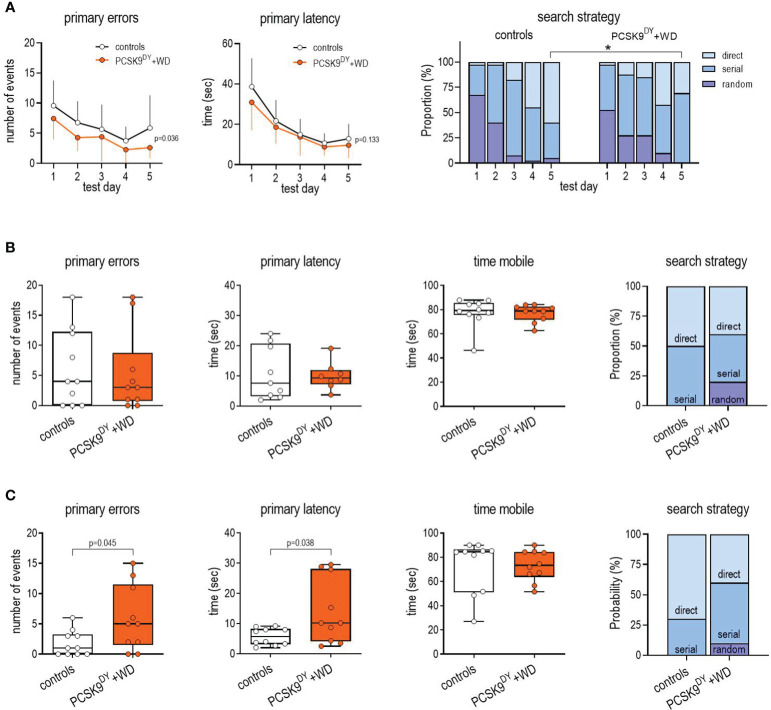
Testing of learning **(A)**, long term memory **(B)** and remote memory **(C)** following the BM approach in C57Bl/6 mice that received a single AAV-PCSK9^DY^ (2x10^11^ vg) injection plus Western diet or in controls only received chow diet. The median is depicted in box blots; the box extends from the 25th to 75th percentiles and the whiskers go down to the smallest value and up to the largest. A 2way-ANOVA was calculated considering the factors time and PCSK9^DY^+WD within the habituation period (**A**; primary errors: time F=8.29, P<0.0001, PCSK9^DY^+WD F=5.29, P=0.036, interaction F=0.328, P=0.858; primary latency: time F=27.5, P<0.0001, PCSK9^DY^+WD F=2.47, P=0.133, interaction F=0.441, P=0.778). A t-test was calculated with or without Welsh correction (dependent on variance homogeneity) when values showed Gaussian distribution in order to depict differences between the two groups considering long term **(B)** and remote memory **(C)**. When values did not show Gaussian distribution, Mann-Whitney test was calculated. For assessing differences in search strategy, a Chi-squared test was calculated; * p< 0.05, n=10 each group.

The BM test was conducted 13-14 weeks after administering AAV-PCSK9^DY^. For this test, the primary errors (number of errors before first contact with the escape hole), secondary errors (number of errors after first contact with the escape hole), primary latency (time needed for first contact with the escape hole), and, finally, the time mobile (total time until entry into the exit) were analyzed. During the training phase, number of primary errors was slightly lower in the PCSK9^DY^+WD mice, while primary latency did not differ (**Figure 6A**). Furthermore, in the PCSK9^DY^+WD mice, the secondary errors were increased, and the exit interaction^time^ as well as the exit interaction^events^ tended to be increased during the training period, respectively, while the mean speed was decreased. No difference was observed in distance travelled and secondary latency (**Supplementary Figure S6**). Friedman testing indicated that the search strategy was time-dependently changing during the 5 days of habituation in the controls (random p<0.001; serial p=0.01, direct p=0.001) as well as in the PCSK9^DY^+WD mice (random p=0.02; serial p=0.516, direct p=0.002). Chi Quadrat testing indicated that the search strategy differed between the two groups only on d5 (p=0.004). Finally, Mann-Whitney-U-test, considering search strategies at d5, further indicated that the direct strategy was higher in the controls than in the PCSK9^DY^+WD mice (p=0.014), while serial was higher in the PCSK9^DY^+WD than in the controls (p=0.004) (**Figure 6A**). Long term memory in the PCSK9^DY^+WD group was not affected as test performance at d6 was similar in both groups of mice. We detected no difference in primary errors, primary latency and mobile time. The search strategy at d6 seems to be different between the two groups, although a statistical analysis (Chi square testing) was not possible due to the too small group size (**Figure 6B**). Other parameters from the BM evaluation regarding long term memory (d6) and remote memory (d17) were not different between the two groups (**Supplementary Figure S7**). The finding that long-term memory was not affected is confirmed by the OPR test as we did not detect any differences in the index preference (**Supplementary Figure S5**). In contrast, significant functional impairments were observed at d17, representing remote memory. Number of primary errors (3.2-fold) and primary latency (2.5-fold) were higher in PCSK9^DY^+WD mice. In addition, the search strategy changed: the direct strategy was reduced, while the serial and random strategy increased, although it must be mentioned here that a statistical evaluation was not possible due to the small group size (**Figure 6C**).

### PCSK9^DY^+WD depict cerebral vascular dysfunction and impaired CBF

**Figure 7:**
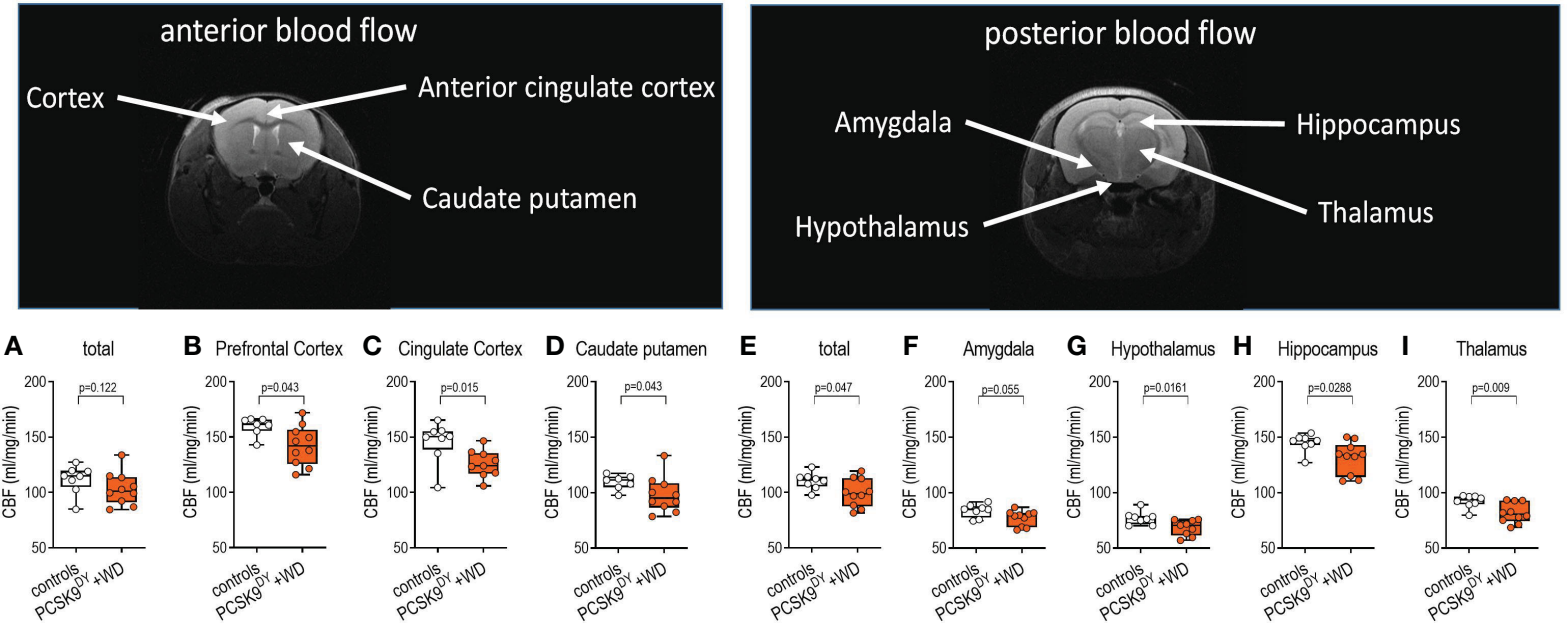
T2W MRI images of brains to determine cerebral blood flow in C57Bl/6 mice in anterior and posterior areas that received a single AAV-PCSK9^DY^ (2x10^11^ vg) injection plus Western diet. Controls only received chow diet. Signal intensity of the T2W MRI images were analyzed to assess the perfusion in total of anterior **(A)** and posterior areas **(E)**, prefrontal cortex **(B)**, cingulate cortex **(C)**, caudate putamen **(D)**, the amygdala **(F)**, hypothalamus **(G)**, hippocampus **(H)**, and thalamus **(I)**. The median is depicted in box blots; the box extends from the 25th to 75th percentiles and the whiskers go down to the smallest value and up to the largest. A t-test was calculated with or without Welsh correction (dependent on variance homogeneity) when values showed Gaussian distribution. When values did not show Gaussian distribution, Mann-Whitney test was calculated; n=10 each group.

After acquiring MRI scans, we analyzed perfusion in the areas of interest. Those areas were defined according to signal intensity of the T2-weighted MRI images. When PCSK9^DY^+WD and control mice were compared, no difference was observed total anterior detected, while the total posterior blood flow was slightly lower in PCSK9^DY^+WD mice (-10%, **Figures 7A, E**). When investigating the blood flow in specific brain areas, we also found no differences in perfusion between the PCSK9^DY^+WD mice and the controls for amygdala or motor prefrontal cortex (**Figures 7B, F**), consistent with the lack of anxiety-like behavior. In contrast, we found decreased perfusion in the cingulate cortex (-17%), caudate putamen (-15%), hippocampus (-10%), and thalamus (-13%) of PCSK9^DY^+WD mice compared to controls (**Figures 7C, D, G–I**). According to TOF angiography of vessel cross sections of the ICA no differences in the lumen area (0.408 ± 0,024 mm^2^ vs. 0.397 ± 0.015 mm^2^; p=0.690) were observed between the two experimental groups.

### PCSK9^DY^+WD leads to hippocampal vascular dysfunction

**Figure 8:**
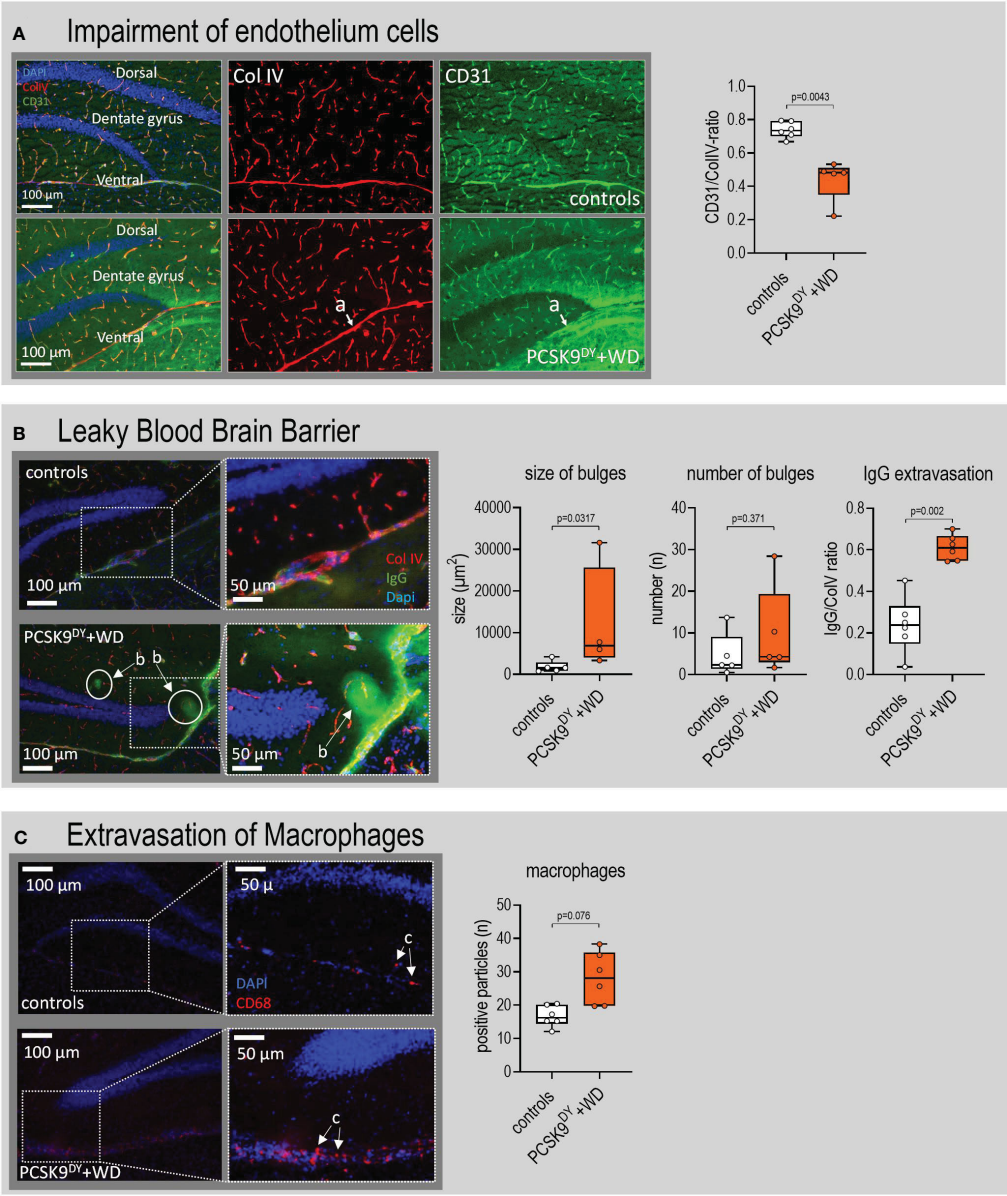
Patchy blood-brain barrier leaks in the hippocampus of C57Bl/6 mice that received a single AAV-PCSK9^DY^ (2x10^11^ vg) injection plus Western diet (WD). Controls only received chow diet. **(A)** Indicates the impairment of endothelium cells in PCSK9^DY^+WD mice as the CD31/Col IV ratio decreased; (a) depicts a medium-sized vessel that lacks CD31 in the ventral hippocampus. **(B)** indicates that BBB is leaky in PCSK9^DY^+WD mice as number of bulges and extravasation of IgG increased in these mice, (b) depicts bulges. In the 50-µm magnifications, the contrast and brightness were increased for better visibility. **(C)** demonstrate extravasation of macrophages in PCSK9^DY^+WD mice as the number of CD68 positive particles was higher compared to controls; (c) depicts macrophages. The median is depicted in box blots; the box extends from the 25th to 75th percentiles and the whiskers go down to the smallest value and up to the largest. A t-test was calculated with or without Welsh correction (dependent on variance homogeneity) when values showed Gaussian distribution. When values did not show Gaussian distribution, Mann-Whitney test was calculated; n=5-6 each group.

**Figure 9:**
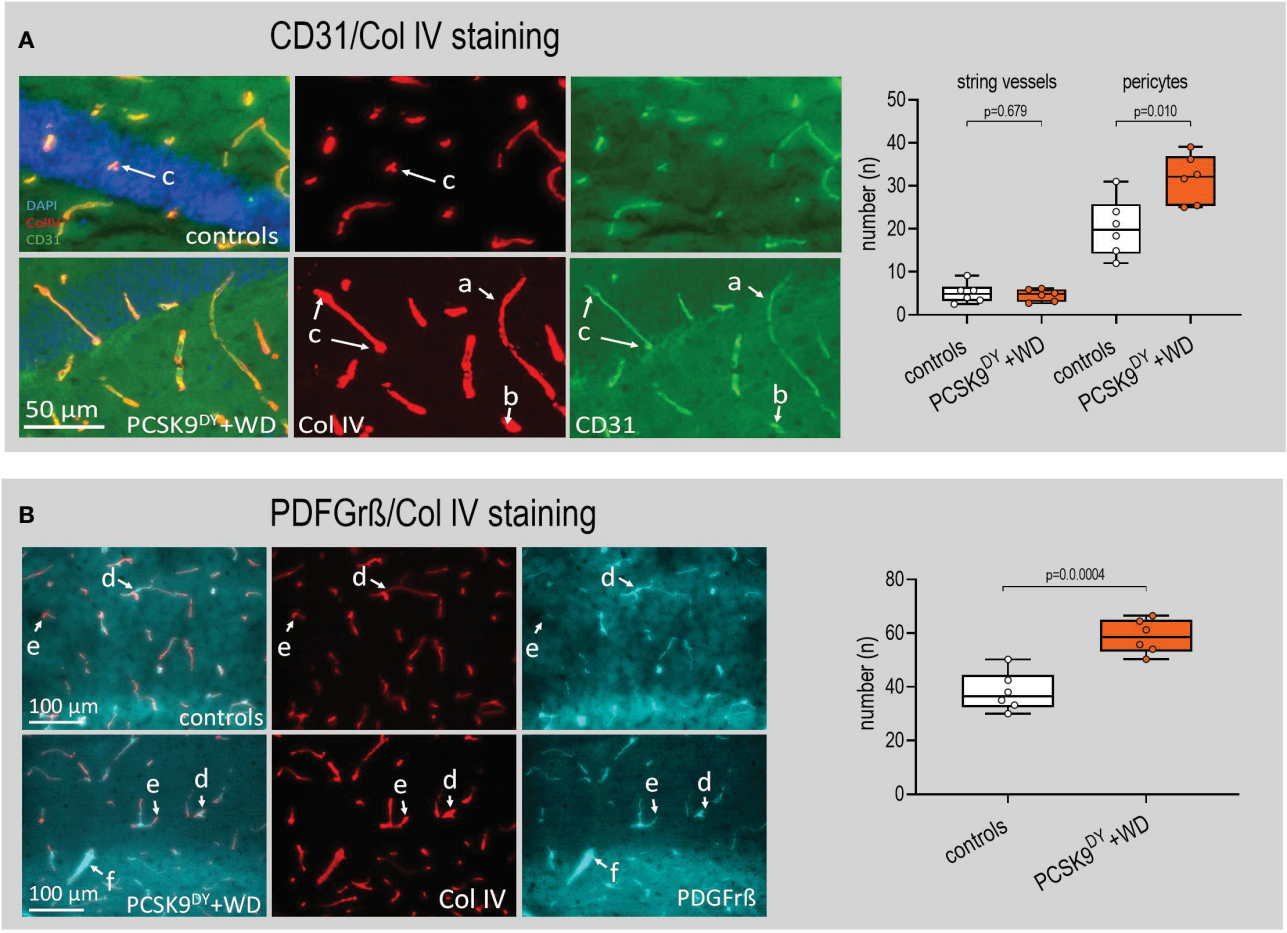
Abundance of string vessels and pericytes hippocampus of C57Bl/6 mice that received a single AAV-PCSK9^DY^ (2x10^11^ vg) injection plus Western diet (WD). **(A)** Hippocampus of PCSK9^DY^+WD mice have a higher number of abnormal small vessels; this analysis considers the string vessels and bulging vessels, probably pericytes; (a) depicts a normal vessel expressing CD31 and Col IV with a regular shape; (b) depicts a Col IV-positive string vessel but no expression of CD31; and (c) depicts pericytes, the vessels have a bulged shape or enlarged Col IV area staining with lower expression of CD31. **(B)** The co-staining of Col IV (red) with PDGFrβ confirms the presence of pericytes in the small vessels of the hippocampus. There is also the presence of abnormal vessels with microaneurysm morphology that lack of PDGFrβ positive pericytes in the hippocampus of PCSK9DY+WD mice; (d) pericyte, (e) microaneurysm, (f) PDGFrβ overexpression. The median is depicted in box blots; the box extends from the 25th to 75th percentiles and the whiskers go down to the smallest value and up to the largest. A t-test was calculated with or without Welsh correction (dependent on variance homogeneity) when values showed Gaussian distribution. When values did not show Gaussian distribution, Mann-Whitney test was calculated; n=5 each group.

Brain slices were immunostained particularly to evaluate changes in the microvascular system of the hippocampus and amygdala. The CD31/Col IV ratio was selectively reduced in hippocampus (-40%, **Figure 8A**) of PCSK9^DY^+WD mice while it was not in the amygdala (**Supplementary Figure S8A**). This indicates changes in microvascular structure. In addition, we found no differences in the CD31/Col IV ratio in cingulate cortex or caudate putamen of PCSK9^DY^+WD or control mice (**Supplementary Figure S9**). Using higher magnifications of the microscopic CD31/Col IV images, we investigated the morphology of the vascular structures in more detail. Whereas there were no changes in the number of string vessels in the hippocampus of the PCSK9^DY^+WD mice, the number of pericytes was indeed increased (+56%) in the hippocampus of the PCSK9^DY^+WD (**Figure 9A**). Those vessels developing Col IV+/CD31 protuberances and dilated vessels with a large Col IV-positive area and small or absent CD31 were classified as pericytes. Again, an increase in pericytes was not seen in the amygdala (**Supplementary Figure S10**). The hippocampus-specific increase in pericytes was further confirmed by PDFGrß staining, as the number of positive cells was increased in PCSK9^DY^+WD mice by 53% (**Figure 9B**). The size, but not the number of patchy blood-brain barrier leaks was higher (2.1-fold) in the PCSK9^DY^+WD mice as detected by infiltration of IgG into the brain parenchyma (**Figure 8B**), but, again, not in the amygdala of PCSK9^DY^+WD-positive mice compared to controls (**Supplementary Figure S8B**). Similarly, CD68 staining (+68%) selectively increased in the hippocampus (**Figure 8C**).

## Discussion

The main findings of our study are that, upon PCSK9^DY^+WD intervention in hyperlipidemic mice, selective hippocampal vascular structural changes are associated with a reduction in blood flow in this brain region, which correlates with impairment of long-term memory in these mice.

As expected, and consistent with our early studies in mice (12, 17), the high-calorie diet causes obesity and higher fat mass. However, free body fluid was also increased in PCSK9^DY^+WD mice as compared to control mice, which may indicate development of congestive heart failure in the study animals as congestive heart failure is typically associated with fluid retention and increased extracellular fluid (26). However, characterizing cardiovascular function in PCSK9^DY^+WD mice was not the focus of this study and development of heart failure has been already reported in the atherosclerotic LDLR/ApoE dko mouse (27).

We previously demonstrated that feeding a high-fat diet for 4 months induced obesity and anxiety behavior but not cognitive impairment in mice (17). Hyperlipidemia is well known to contribute significantly to the factors of obesity-induced cognitive dysfunction (1, 28, 29). In the study mentioned above (17), we did not measure plasma lipids, but in a further study we also performed in high-fat diet-fed Bl6 mice, TC was increased only by a factor of 0.35 in the HFD-fed mice, whereas TG was unaffected (30). Instead of significantly extending the feeding duration or even using older animals, we indeed induced hyperlipidemia via the PCSK9^DY^+WD approach as TC was increased by a factor of 10 and TG by a factor of 6. In line with other studies (10–12), we found that as a result of the PCSK9^DY^+WD intervention not only hyperlipidemia but also changes characteristic of atherosclerosis developed, such as a manifest plaque burden and increased plasma concentrations of various circulating cytokines. Using RNAseq analysis, we showed for the first time in PCSK9^DY^+WD-positive mice that the expression of genes of inflammatory signaling pathways was significantly higher in the aortic segments than in lean controls. Based on KEGG pathway annotation analyses we further confirmed for the PCSK9^DY^+WD-dependent atherosclerosis model that various pathways which are upregulated in other atherosclerotic mouse models, including natural killer cell-mediated cytotoxicity (31, 32), cell adhesion molecules (CAMs) (33–35), hematopoietic cell lineage (36), B cell-receptor signaling pathway (37, 38), cytokine-cytokine receptor interaction (38), primary immunodeficiency (39), and osteoclast differentiation (40), were involved. Interestingly, of the top ten most upregulated genes in aortas of PCSK9^DY^+WD mice, six are associated with atherosclerosis in experimental and clinical studies: Cd5L, which is expressed mostly by macrophages in inflamed tissues (41), is upregulated in stable plaques (42, 43). Gene and protein expression of matrix metallopeptidase 12 (MMP12) is increased in aortas from ApoE-deficient mice (44). The proinflammatory IL-1-alpha (IL-1α) is enhanced in atherosclerosis (45). Elovl3 (participating in the production of saturated and monounsaturated very long-chain fatty acids) was downregulated by the drug PX-478, thereby also reducing atherosclerosis (46). Secreted phosphoprotein 1 (SPP1, osteopontin, which has been implicated as an important factor in remodeling processes) is increased in atherosclerosis (47, 48). C-type lectin domain family 1 member B (Clec1b) levels were associated with an increased risk of carotid plaque and was assumed to predict subclinical atherosclerosis (49). Of the top ten downregulated genes, atrial natriuretic peptide (ANP) was found to be the one downregulated to the greatest degree, which is in accordance with other studies showing that aortas with severe atherosclerosis were less sensitive to ANP (50). Moreover, Hamp, which codes for hepcidin, was downregulated in PCSK9^DY^-treated mice and also lower in liver and spleen of ApoE-deficient mice (51). Hepcidin has an impact on iron homeostasis in atherosclerosis (52). It has already been shown that aortic aneurysms are associated with increased recruitment of monocytes to aneurysms, and this is promoted by upregulating I-CAM, V-CAM, MCP1, and MMP2 (25) and over-expressing the matrix metalloproteinase family (MMP) (53), all contributing to vessel remodeling. All these genes were upregulated in aortas of PCSK9^DY^-positive mice, too. As Sirtuin 1 (Sirt1) has been shown to be important to cardiovascular disease, inflammation, cognition, hippocampal function, cerebral blood flow and learning and memory (54, 55) and Sirt1 and Sirt6 suppress inflammation, Sirt1 prevents apoptosis and cell death, Sirt3 inhibits oxidative stress and Sirt2 regulates LDL cholesterol by inhibiting PCSK9 and increases LDL receptors on the cell surface of hepatocytes (56), we also determined expression levels of sirtuins in aorta. mRNA seq analysis revealed no differences in sirtuins (Sirt) in aortas of PCSK9^DY^/WD mice. On the one hand this is in contrast to findings showing that Sirt1 expression was reduced in human atherosclerotic plaques and vascular smooth muscle cells (VSMCs) but on the other hand in the same publication no reduced Sirt1 expression could be detected in aortas of ApoE deficient mice, which we then also saw in our study in the PCSK9^DY^/WD based atherosclerosis mouse model (57). Thus, it might be worthwhile for follow-up studies to investigate Sirt1 expression not globally in aortas, but rather in plaques and VSMCs, but even more so in cerebral tissue, in order to more clearly investigate the relevance of these pathways in the PCSK9^DY^/WD model. Taken together, our results clearly support that the notion that the PCSK9^DY^+WD approach is a promising model of hyperlipidemia and aortic dysfunction to further investigate whether microvascular alterations affect cognitive behavior.

Regarding ApoE-deficient mice, which are an established experimental model of atherosclerosis, spatial learning and long-term memory was impaired in the Morris water maze (MWM) (58, 59). However, other behavioral studies on ApoE-deficient mice did not reveal any differences (60, 61). These conflicting findings have been related to differences in genetic background, housing conditions, diets, and inconsistencies in behavioral tasks, particularly in the MWM approach (62). Since mice are more physiologically adapted to dry land tasks, this confers an advantage to the BM task to better elucidate certain learning/navigation behaviors. Moreover, the BM task is undoubtedly less stressful than the MWM (63). Accordingly, we favored the use of the BM approach. Based on the results of the BM test that we collected, we conclude firstly that PCSK9^DY^-WD mice tend to detect the escape hole more easily during acquisition (lower primary errors and primary latency), but ultimately take longer to escape from the platform (higher secondary errors and higher latency). This could be due to a physical impairment to escape into the hole, but we did not gain circumstantial evidence for this in this study. Furthermore, it could be attributed to a greater fear of entering the hole, but the EPM data show no signs of fear. As well based on the results from the acquisition phase, we further conclude that spatial learning is somewhat impaired in the PCSK9^DY^+WD mice as the search strategy was affected, yet the PCSK9^DY^+WD showed a lower extent of direct search strategy especially at day 5. Second, because the primary error and primary latency were increased compared to the controls at d17 but not at d6, we conclude that remote memory is impaired in PCSK9^DY^+WD positive mice, whereas simple long-term memory is unchanged. Third, comparing the performance in the experiments on d6 and d17, it is evident that the controls (P=0.0312, paired T-test) but not the PCSK9^DY^+WD positive mice (P=0.796, paired T-test) performed better on d17 than on d6. This could be interpreted to mean that we observe memory consolidation in the controls but not in the verum animals. This assumption also seems to be supported by the fact that the direct search strategy also increased from d6 to d17 in the controls, whereas it remained rather unchanged in the PCSK9^DY^+WD positive mice.

Hyperlipidemia could cause cognitive deficits but also pathologic capillary changes, which are shown here. Wang (64) attributed cognitive defects in response to HF diet to rapid hippocampal oxidative stress and alterations in synaptic plasticity and Zhuang (65) to alterations in hippocampal neuroplasticity as changes in microglial phenotype appeared that are accompanied by a remarkable increase in cellular lipid accumulation. Conversely, intermittent fasting not only exhibited improved long-term memory but also increased the number of BrdU-labeled cells and neuroblasts in the hippocampus and expression of the longevity gene Klotho (66). We investigated blood flow in these areas to answer the question of whether the cognitive deficits that we and others have observed are related to a reduction in blood flow in these areas: indeed, blood flow was reduced in the hippocampus of mice with long-term memory deficits. This confirms our earlier findings that normal cognitive function in obese-only animals is accompanied by unaltered hippocampal blood flow (17). Blood flow was also reduced in cingulate cortex and caudate putamen of PCSK9^DY^+WD-treated animals, which fits perfectly to impaired spatial learning as the cingulate cortex is related to decision-making ability (67) and the caudate putamen is important for learning tasks (68).

As anxiety behavior has been correlated with amygdala processes (69), our observation seems to be consistent as we did not observe anxiety behavior or reduced blood flow in the amygdala of PCSK9^DY^+WD-positive mice, while Huber et al. attributed the increased anxiety behavior of HF-fed mice to the reduced blood flow in the amygdala (17). Why we did not observe anxiety or altered blood flow in the amygdala is unclear, as the PCSK9^DY^ mice indeed became obese when fed with WD. However, the extent of the obesity was much less pronounced than in the Huber study (17), with only a third of the weight gain. It may also be speculated that the age of mice, virus administration, or differences in the diet could be causative.

We next sought to understand the causes for the reduced blood flow in PCSK9^DY^+WD mice. Obesity and diabetes were claimed to be bad predictors of brain microvasculature stability (70). While published studies (and those in the present study being confirmatory) in the PCSK9^DY^-based model mainly found a dysfunction of larger vessels (especially the aorta), we show, moreover, for the first time by immunohistological analysis microvascular brain vessel dysfunction. The CD31/Col lV ratio was lower in hippocampus of PCSK9^DY^+WD mice, which might be associated with more string vessels or pericytes. Mice with cognitive impairment typically show string vessel formation (71). CD31 as well as PDFGrß staining clearly indicate a selective increase in pericytes in hippocampus. Pericytes are unique, multi-functional mural cells localized at the abluminal side of the perivascular space in microvessels. The role of capillary pericytes in CBF controls has been intensively discussed and probably depends on subclassification according to their topology, morphology, and protein expression levels (72). Consensus seems to be that in response to different neurotransmitters, pericytes normally dilate capillaries and increase local CBF. However, in pathological conditions such as ischemic stroke and AD, brain capillaries are constricted by pericytes, thus lowering CBF (72). Using TOF angiograms, we measured vessel diameters of the ICA. However, we failed to demonstrated increased contractility in the PCSK9^DY^-treated mice. We attribute this to the fact that pericytes are located on the microvessels, which are distributed on the precapillary arterioles, capillaries, and postcapillary venules (72). Thus, a more sensitive technique such as the microCT imaging should be used to better determine brain vasculature (73).

Cerebral aneurysm is also present in vascular dementia (7), cerebral amyloid angiopathy (7, 74), and AD (74, 75). Such events exacerbate cerebrovascular phenomena such as microbleeds and consequently impair neurovascular functions. The development of microaneurysms has been reported in metabolic and cardiovascular diseases (76, 77) and has been described in particular in the retinal microvasculature of diabetes models (78–80). The burden of atherosclerosis promotes the progression of retinopathy and the development of microaneurysms (81). The genetically/diet-induced atherosclerosis models also promote the development of aneurysms and periaortic microaneurysms (25), which increase with age (82). Lesions also develop in the elastin layer of the vessels in ApoE-ko, which develop into periaortic pseudomicroaneurysms (83). Accumulation of CD68^+^ macrophages in cerebral vessels is a sign of wall vessel damage in cerebral aneurysms (84). This phenotype has not been previously described in the PCSK9^DY^ model and we now found that CD68 staining is increased in hypothalmus of these mice. Very recently, De Shepper et al. showed in APP^NF-L^ mice (serving as an experimental model for AD) a pattern of CD68 expression that is in line with our findings. Moreover, they showed, on the one hand, how the perivascular macrophages respond to deposition of amyloid beta in the perivascular space and, on the other, that those macrophages activate the microglia via SPP1 that triggers synaptic phagocytosis in the hippocampus (85). Against the background of our study, the previously mentioned findings seem worthy of being highlighted, as we found upregulation of SPP1 in the aorta of PCSK9^DY^+WD mice in RNAseq analysis. However, we acknowledge that we have not yet performed RNAseq or, even better, single-cell RNAseq analyses in the hippocampus to demonstrate organ- and cell-specific gene regulation in dependence of PCSK9^DY^ intervention. Despite these results, it seems unclear whether we also have evidence of microaneurysms and microbleeds. We cannot draw conclusions about the diameter of the vessels from our histological images as they did not show enlargement of the vessel lumen. We did find that infiltration of IgG into the brain parenchyma was higher in the PCSK9^DY^+WD mice. This extravasation of the blood component IgG might constitute a surrogate parameter for microbleeds; however, it would have been helpful to detect blood as confirmation. Thus, we defined the IgG increase more in terms of patchy blood-brain barrier (BBB) leaks (86). BBB breakdown was recently found to be an early biomarker of cognitive dysfunction (87).

In summary, we conclude that atherosclerosis causes microvascular dysfunction in PCSK9^DY^+WD mice, reducing blood flow in the hippocampus, which could explain the cognitive dysfunction in these mice. Consequently, use of a PCSK9^DY^+WD mouse model offers a valuable method for further research into pharmacological interventions.

## Data availability statement

The datasets presented in this study can be found in online repositories. The names of the repository/repositories and accession number(s) can be found below: https://www.ncbi.nlm.nih.gov/geo/query/acc.cgi?acc=GSE247400, GSE247400.

## Ethics statement

The animal study was approved by Ministerium für Landwirtschaft, ländliche Räume, Europa und Verbraucherschutz des Landes Schleswig-Holstein, Germany. The study was conducted in accordance with the local legislation and institutional requirements.

## Author contributions

LH: Data curation, Formal analysis, Investigation, Methodology, Visualization, Writing – original draft. FR: Data curation, Investigation, Methodology, Writing – original draft, Funding acquisition. EP: Data curation, Methodology, Writing – review & editing. OW: Data curation, Methodology, Writing – review & editing. J-BH: Writing – review & editing, Conceptualization. FS: Writing – review & editing, Methodology. ÜÖ: Methodology, Writing – review & editing. JL: Methodology, Writing – review & editing. IS: Methodology, Writing – review & editing. ZA: Data curation, Formal analysis, Methodology, Visualization, Writing – review & editing. CK: Formal analysis, Software, Visualization, Writing – review & editing. KK-V: Funding acquisition, Methodology, Writing – review & editing. UM: Methodology, Writing – review & editing. SH: Methodology, Writing – review & editing. RB: Conceptualization, Funding acquisition, Supervision, Writing – review & editing. MS: Methodology, Writing – review & editing. OM: Methodology, Writing – review & editing. WR: Conceptualization, Data curation, Formal analysis, Funding acquisition, Investigation, Methodology, Project administration, Resources, Supervision, Validation, Visualization, Writing – original draft.
