# Supplementary material for: Incidence of microvascular dysfunction is increased in hyperlipidemic mice, reducing cerebral blood flow and impairing remote memory
Source: Front Endocrinol (Lausanne). 2024 Feb 26;15:1338458. doi: 10.3389/fendo.2024.1338458 (PMC10925718; doi:10.3389/fendo.2024.1338458)
Supplement: Supplementary file 1 [file DataSheet_1.pdf]

Tab s1: primary antibodies

antibody	company	#	dilution
rat anti-CD31	Abcam	2161026	1:100
goat anti-Col IV	Abcam	2082646	1:100
rat anti-CD68	Abcam	322219	1:100
rabbit anti-PDFGrß	Abcam	2162497	1:100

Tab s2: Secondary antibodies

antibody	company	#	dilution
donkey anti-rat Alexa 488-conjugated	Abcam	2535794	1:200
donkey anti-goat Alexa 555-conjugated	Abcam	2532853	1:200
donkey anti-rat Cy3-conjugated	Abcam	2340666	1:200
donkey anti-mouse IgG Alexa 488-conjugated	Abcam	2535792	1:200

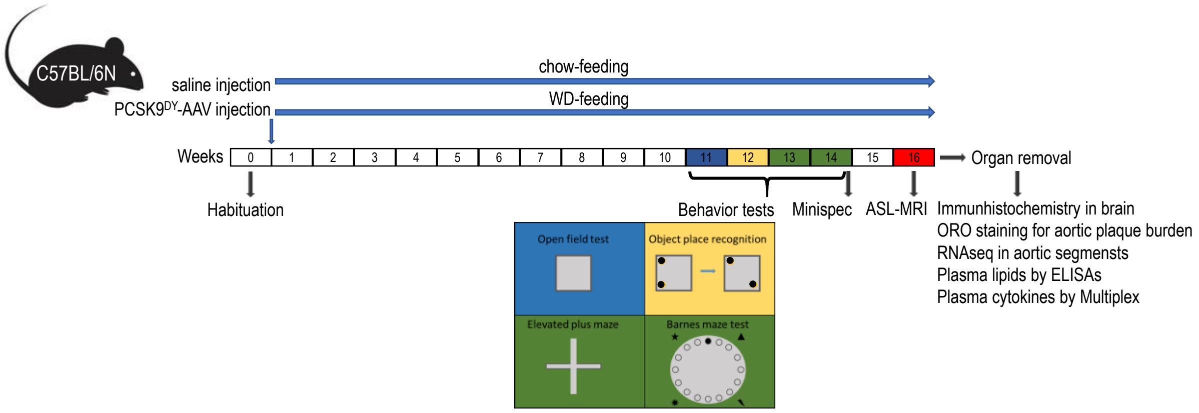

Fig S1: study protocol

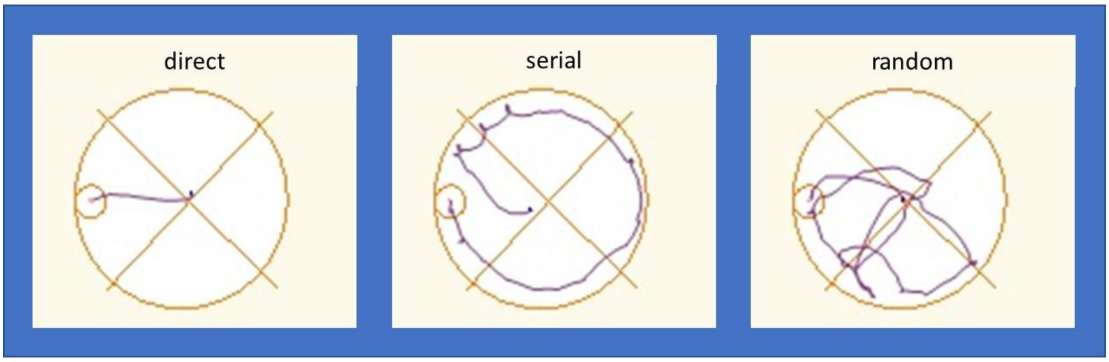

Fig S2: Different search strategies (direct, serial or random) in Barnes Maze test.

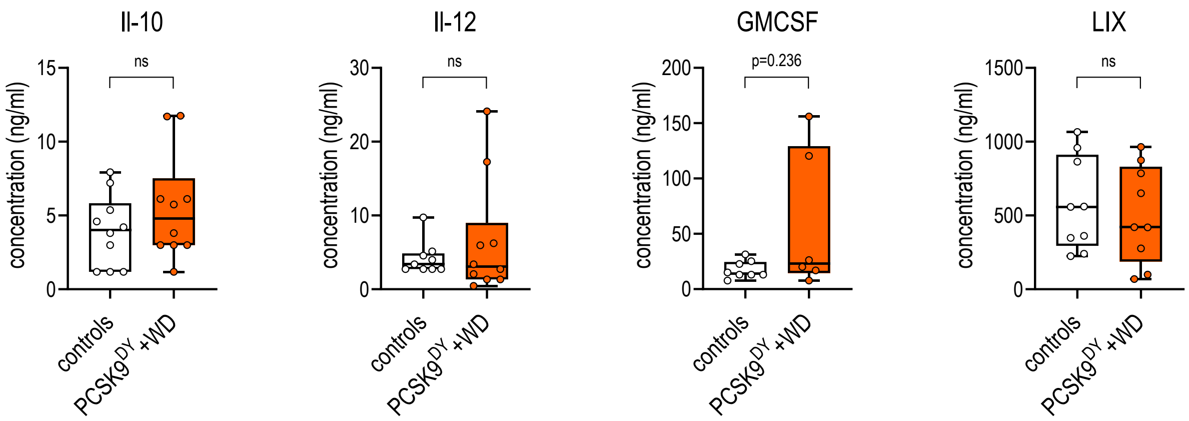

Fig. S3: Plasma concentration of various cytokines in C57Bl/6 mice that received a single AAV-PCSK9^{DY} (2x10^{11} vg) injection plus Western diet. Controls only received chow diet. The median is depicted in box blots; the box extends from the 25th to 75th percentiles and the whiskers go down to the smallest value and up to the largest. A t-test was calculated with or without Welsh correction (dependent on variance homogeneity) when values showed Gaussian distribution. When values did not show Gaussian distribution, Mann-Whitney test was calculated; n=10 each group.

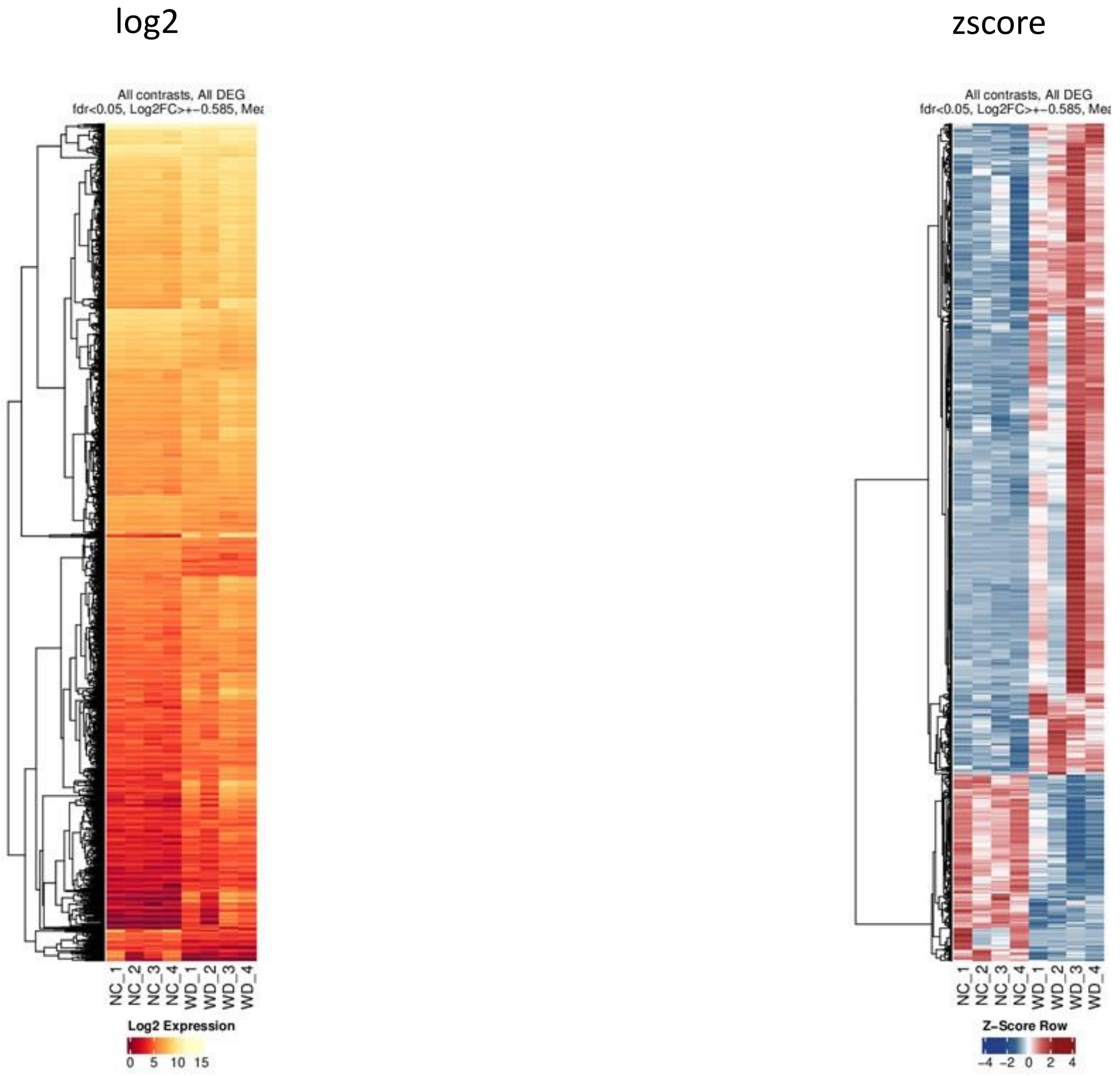

Fig S4: DEG and Z-score: Significantly differently gene expression based on a combination of absolute expression (basemean), divergence (log2 fold change, log2fc), and significance (adjusted pvalue, padj. P-value denotes the probability that results are significant (e.g., significantly different based on absolute counts (basemean) and difference (log2fc)). The p-value identifies genes/peaks with reasonable expression rates and fold changes that show reproducible counts between replicates of conditions. Padj, FDR, Q-value: These terms are not consistently defined, but generally denote a p-value after applying multiple testing corrections (e.g., Benjamini-Hochberg) in order to control for the number of false positives. These are limited to 5% if padj is set to 0.05. Thus, if there are 20 candidates with padj < 0.05, only one of them will be a false positive. If the p-value is used without correction, the number of false positives is statistically unknown. Intuitively, the multiple testing correction is done because it is easier to find the only 10 correct candidates among 100 genes than among 1000000 genes because every test increases the chance to get a false positive. Log2FC: log2(mean(counts condition1) / mean(counts condition2)); Z-score: This normalization method can meaningfully compare widely different distributions. The z-score is defined as the number of standard deviations that a value is above or below the mean of all values.

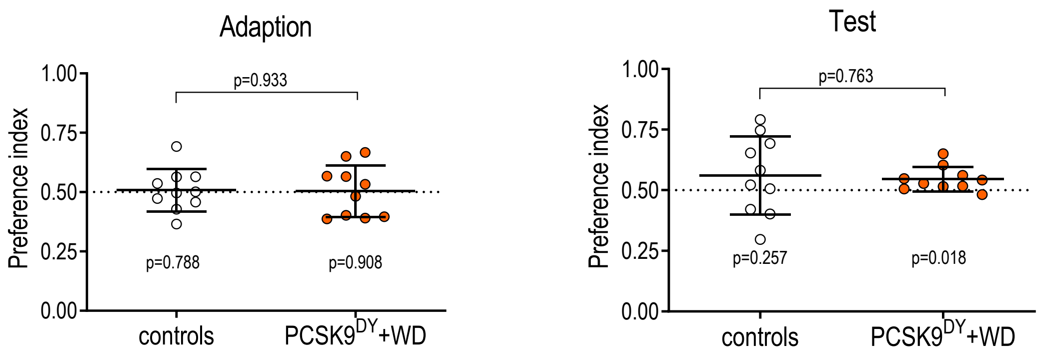

Fig S5: Objective replace test in C57Bl/6 mice that received a single AAV-PCSK9^{DY} (2x10^{11} vg) injection plus western diet. Controls only received chow diet. Values are depicted as means ± SD. The p-value below symbols indicate whether the preference index was different from 0.5, which was calculated by a one group t-test. This p-value thus indicates whether the mouse particularly explores one of the objects. To test the difference between the two groups, a t-test was conducted; n=10.

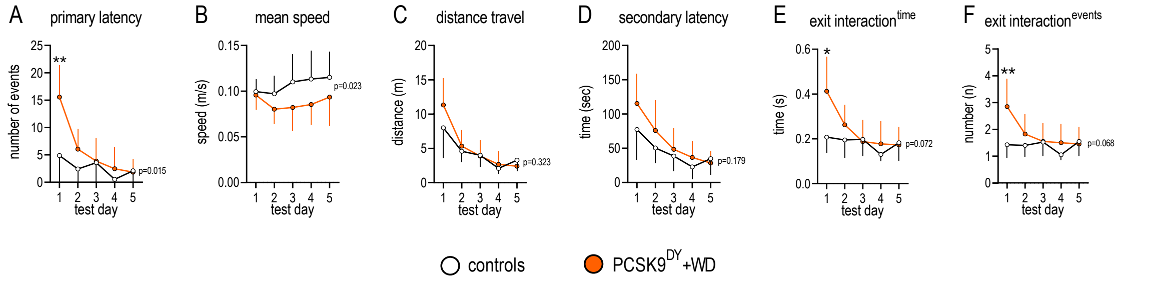

Fig. S6: Additional evaluation parameters of the Barnes Maze test during the 5-day habituation phase of PCSK9^{DY}+WD mice or controls. A way ANOVA test was calculated considering the two factors time and PCSK9^{DY}+WD treatment. A Sidak´s multiple comparisons test was calculated for comparing differences between specific time points. A: primary latency (numbers of errors after the first interaction with the escape hole), time F=2.21, P<0.0001, PCSK9^{DY}+WD F=1.18, P=0.013, interaction F=4.69, p<0.0001; B: mean speed (mean speed of the mice movement through the maze), time F=1.87, P=0.144, PCSK9^{DY}+WD F=6.21, P=0.023, interaction F=1.46, p=0.221; C: distance travel (distance that the mice has moved from the center of the maze until the end of the test), time F=42.2, P<0.0001, PCSK9^{DY}+WD F=1.033, P=0.323, interaction F=3.12, p=0.020; D: secondary latency (during the learning phase, this is the time the mice required to solve the test, from second 0 until they enter into the escape hole), time F=44.5, P<0.0001, PCSK9^{DY}+WD F=1.957, P=0.179, interaction F=4.02, p=0.005; E: exit interaction^{time} (this is the total time the mice had been exploring the escape hole, regardless of whether the mice enters or not), time F=18.6, P<0.0001, PCSK9^{DY}+WD F=3.65, P=0.072, interaction F=9.73, p<0.0001; F: exit interaction^{events} (this is the total number of interactions with the escape hole, regardless of whether the mice enters or not), time F=11.5, P<0.0001, PCSK9^{DY}+WD F=3.77, P=0.068, interaction F=10.1, p<0.0001; means±SD, n=10.

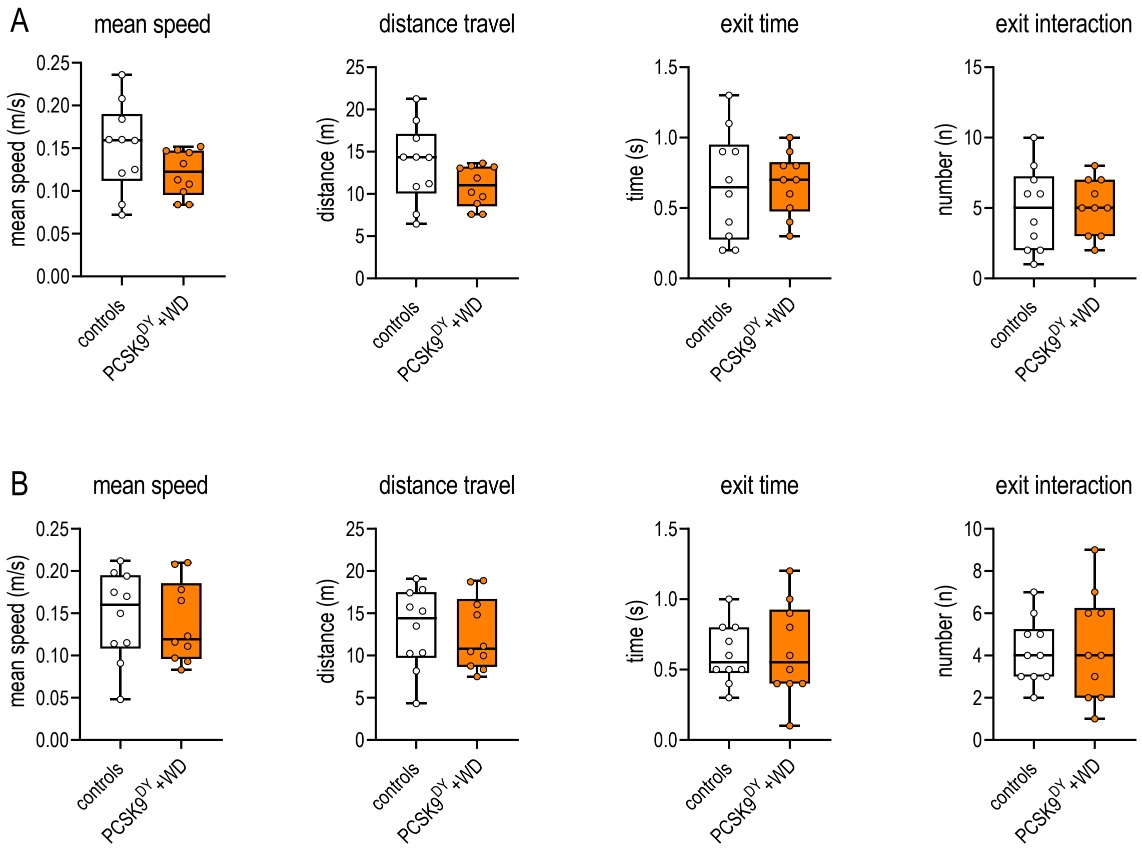

Fig. S7: Additional evaluation parameters of the Barnes Maze test at d6 (A, indicating long term memory) or at d17 (indicating remote memory) of PCSK9^{DY}+WD mice or controls. The median is depicted in box blots; the box extends from the 25th to 75th percentiles and the whiskers go down to the smallest value and up to the largest. A t-test was calculated as values showed Gaussian distribution. When values did not show Gaussian distribution, n=10 each group

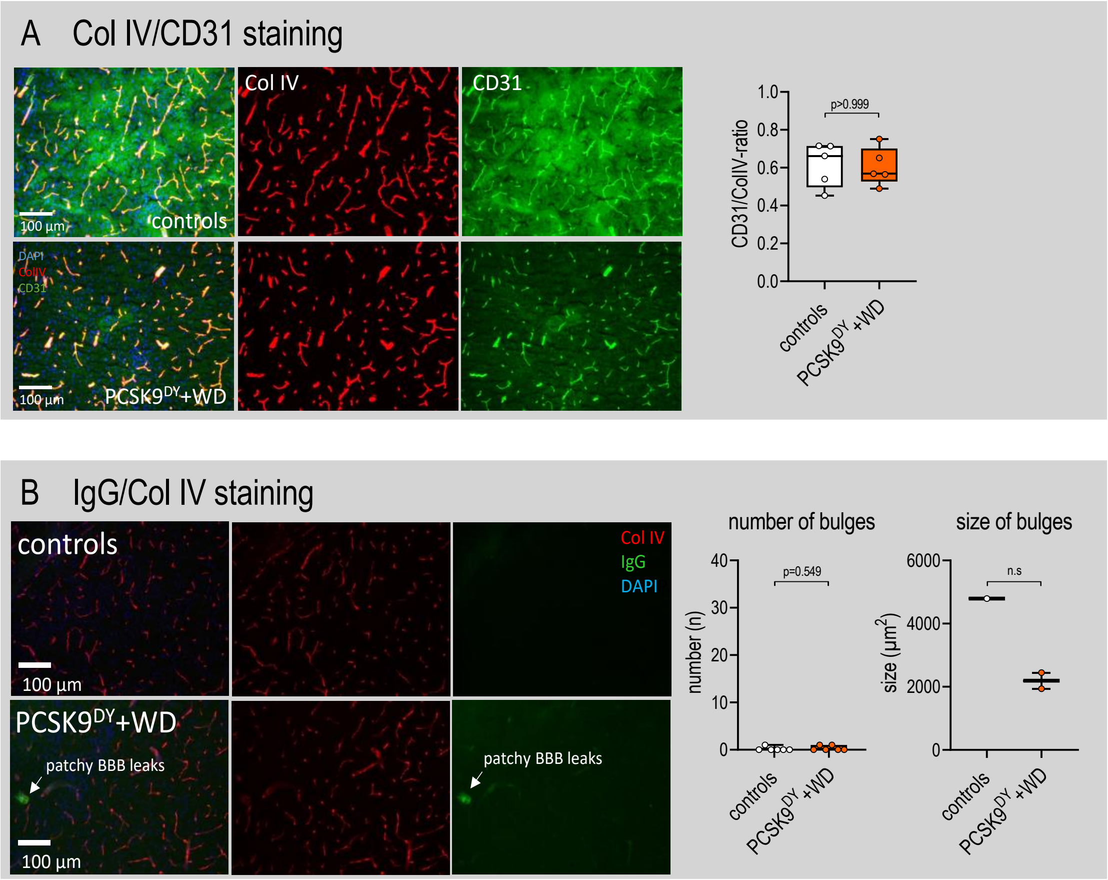

Fig. S8: Integrity of vascular endothelium cells and blood brain barrier in the amygdala of C57Bl/6 mice that received a single AAV-PCSK9^{DY} ($2x10^{11}$ vg) injection plus Western diet (WD). Controls only received chow diet. Fig A illustrates Col IV/CD31 staining as a tool to evaluate vascular endothelium cells. Fig. B illustrate IgG staining to identify whether BBB is leaky. The median is depicted in box blots; the box extends from the 25th to 75th percentiles and the whiskers go down to the smallest value and up to the largest. A t-test was calculated with or without Welsh correction (dependent on variance homogeneity) when values showed Gaussian distribution. When values did not show Gaussian distribution, Mann-Whitney test was calculated; n=5-6 each group.

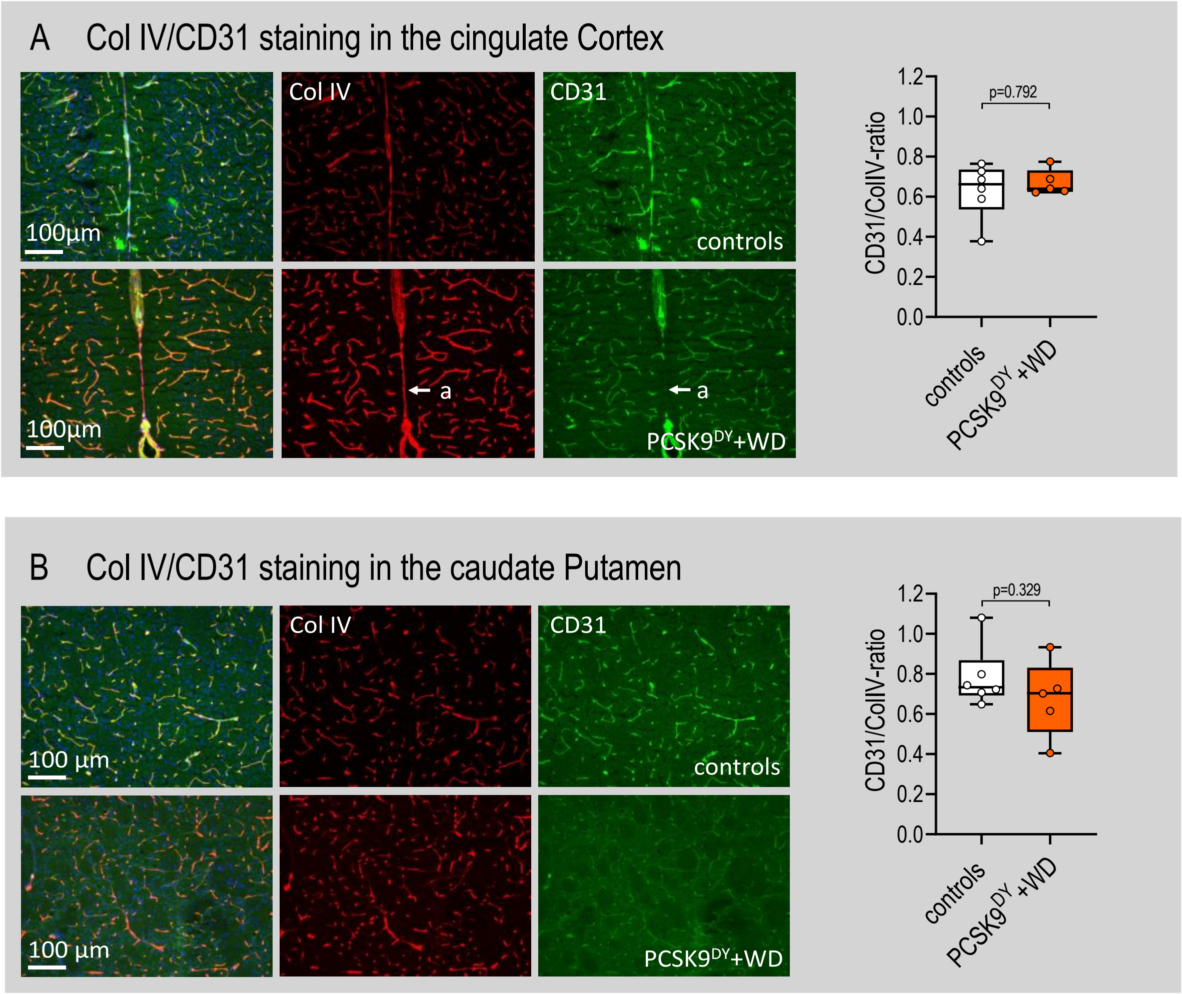

Fig. S9: CD31/Col IV ratio in cingulate cortex (A) and caudate putamen (B) of C57Bl/6 mice that received a single AAV-$PCSK9^{DY}$ ($2x10^{11}$ vg) injection plus western diet. Controls only received chow diet; (a) Venules pointed with the arrows have segments lacking CD31. The median is depicted in box blots; the box extends from the 25th to 75th percentiles and the whiskers go down to the smallest value and up to thxe largest. A t-test was calculated with or without Welsh correction (dependent on variance homogeneity) when values showed Gaussian distribution. When values did not show Gaussian distribution Mann-Whitney test was calculated; n=5 each group.

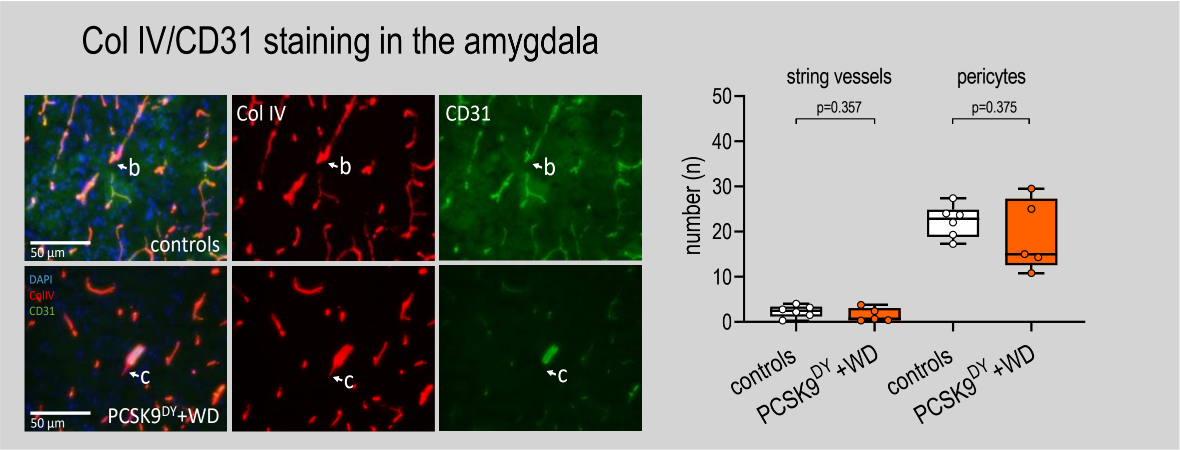

Fig. S10: Abundance of string vessels and pericytes in amygdala of C57Bl/6 mice that received a single AAV-PCSK9^{DY} (2x10^{11} vg) injection plus Western diet (WD). Fig A: Hippocampus of PCSK9^{DY}+WD mice have a higher number of abnormal small vessels; this analysis considers the string vessels and bulging vessels, probably pericytes; (b) depicts a Col IV-positive string vessel but no expression of CD31; and (c) depicts pericytes, the vessels have a bulged shape or enlarged Col IV area staining with lower expression of CD31. The median is depicted in box blots; the box extends from the 25th to 75th percentiles and the whiskers go down to the smallest value and up to the largest. A t-test was calculated with or without Welsh correction (dependent on variance homogeneity) when values showed Gaussian distribution. When values did not show Gaussian distribution, Mann-Whitney test was calculated; n=5 each group.
